# Identification of Novel Human Breast Carcinoma (MDA-MB-231) Cell Growth Modulators from a Carbohydrate-Based Diversity Oriented Synthesis Library

**DOI:** 10.3390/molecules21101405

**Published:** 2016-10-20

**Authors:** Elena Lenci, Riccardo Innocenti, Alessio Biagioni, Gloria Menchi, Francesca Bianchini, Andrea Trabocchi

**Affiliations:** 1 Department of Chemistry “Ugo Schiff”, University of Florence, Via della Lastruccia 13, 50019 Sesto Fiorentino, Florence, Italy; elena.lenci@unifi.it (E.L.); riccardo.innocenti@stud.unifi.it (R.I.); gloria.menchi@unifi.it (G.M.); 2 Department of Clinical and Experimental Biomedical Science “Mario Serio”, University of Florence, Viale Morgagni 50, 50134 Florence, Italy; alessio.biagioni@unifi.it

**Keywords:** heterocycles, sugars, chemical diversity, cancer

## Abstract

The application of a cell-based growth inhibition on a library of skeletally different glycomimetics allowed for the selection of a hexahydro-2*H*-furo[3,2-*b*][1,4]oxazine compound as candidate inhibitors of MDA-MB-231 cell growth. Subsequent synthesis of analogue compounds and preliminary biological studies validated the selection of a valuable hit compound with a novel polyhydroxylated structure for the modulation of the breast carcinoma cell cycle mechanism.

## 1. Introduction

**Figure 1:**
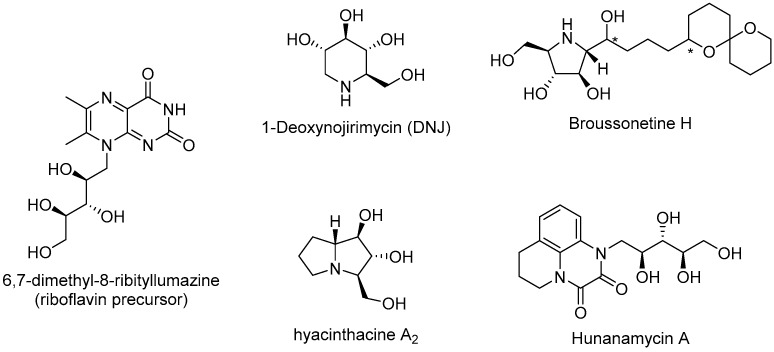
Representative small molecule polyhydroxylated natural products.

In the last 25 years, target-based drug discovery has become a paradigm in both the pharmaceutical industry and in academia. However, considering that it has proved hard to increase the number of truly innovative new drugs, the interest towards phenotypic screening of large small molecule libraries is growing fast [1,2]. In this context, Diversity-Oriented Synthesis (DOS) [3,4,5] has proved to be very useful for the creation of high quality chemical libraries for early-stage drug discovery programmes. In fact, it operates to generate the maximum skeletal and stereochemical diversity by using forward synthetic analysis and complexity-generating reactions [6,7]. Recently, natural product structures have proved to be optimal starting materials for DOS strategies [8,9,10], thanks to their structural feasibility to generate compounds in the medicinally relevant chemical space and following Lipinski’s rules [11]. In particular, carbohydrates can be exploited for the achievement of skeletal diversity, taking advantage of their conformational constraints and the high density of polar groups. Although the application of carbohydrates in traditional combinatorial chemistry has been reported since the nineties [12,13,14], they remain rather underexplored in DOS strategies, mainly because of their need for transitional protection/deprotection stages [15,16,17,18,19,20,21]. In this context, we have reported the use of d-mannose in combination with glycine-derived aminoacetaldehyde for the synthesis of an array of novel skeletally different polyhydroxylated nitrogen-containing scaffolds [22]. The relevance of these compounds was attested by the widespread distribution of polyhydroxylated nitrogen-containing natural products in plants and microorganisms (Figure 1). In particular iminosugars [23,24,25], pyrrolidine [26,27], and pyrrolizidine alkaloids [28,29,30,31,32] occupy relevant positions in biomedical issues, for their potential in the development of new treatments against cancer, infective diseases, diabetes, and metabolic disorders [33,34,35].

DOS small molecule libraries are often applied in phenotypic screening or chemical genetics studies in search of hit compounds capable of inducing a desired phenotype, such as toxicity to bacteria or the ability to selectively kill cancer cells [36,37,38,39]. This ‘forward pharmacology’ process is particularly successful for lead discovery in those complex disorders, such as cancer and neurological and infective pathologies, where multiple targets are involved and/or physiopathological pathways have yet to be discovered [40,41,42]. Just to give some examples, nifedipine, nimodipine, and other calcium channel antagonists were actually discovered by the application of phenotypic screens in search for compounds able to induce vasodilatation and blood pressure reduction [43,44]. In recent years, different phenotypic screening methods have been developed, especially in the pharmaceutical industries, were robotic and miniaturized technologies allow for rapidly screening large chemical collections. Different phenotypes can be taken into account, such as the capability of killing pathogens or cancer cells, or the modulation of autophagy and apoptosis, and cell cycle mechanisms are often the most studied ones [45]. In particular, we reasoned to apply a cell-based phenotypic screening in search of compounds inducing a significant cell growth arrest on MDA-MB-231 cell lines. This cell line is a simple model system for the study of the triple-negative breast cancer (TNBC). This type of cancer shows a major tendency toward early metastasis [46,47], and does not respond to hormonal chemotherapy, as it lacks the three main molecular targets, the estrogen receptor (ER), the progesterone receptor (PR), and the human epidermal growth factor receptor (HER-2/Neu) [48,49,50]. Therefore, the development of new treatments against such type of cancer, which accounts for 15% of all type of breast carcinomas, is highly needed. Although further research is still necessary, some preliminary evidence about the ability of iminosugars to inhibit breast cancer cell growth has recently appeared in the literature. In particular, different types of pyrrolidinic compounds have shown significant cell growth inhibition in breast tumoral cell lines, such as T-470 [51] and MCF-7 line [52]. In addition, iminosugar-ferrocene conjugates proved to inhibit MDA-MB-231 breast cancer cells proliferation [53].

In this work we present the application of a MDA-MB-231 cell-based growth inhibition assay on a library of skeletally different glycomimetics, and the follow-up synthesis of compounds containing the same scaffold as of selected molecules for preliminary biological studies aiming to identify a valuable hit compound for the modulation of breast carcinoma cell cycle mechanism.

## 2. Results and Discussion

**Figure 2:**
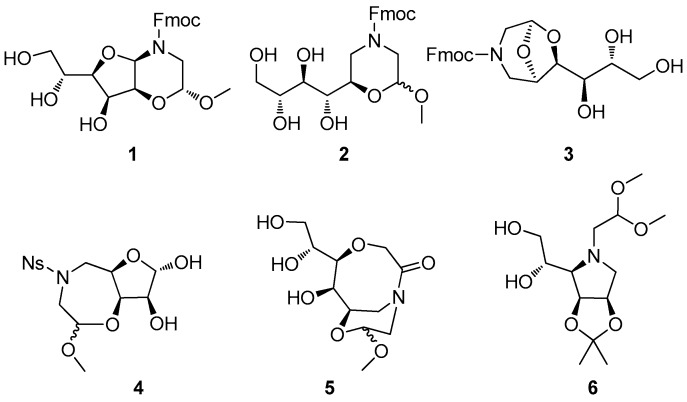
Polyhydroxylated nitrogen-containing scaffolds derived from d-mannose and glycine-derived aminoacetaldehyde.

The diversity-oriented synthesis of six polyhydroxylated nitrogen-containing scaffolds was achieved by the combination of two building blocks, easily obtained from d-mannose, with glycine-derived aminoacetaldehyde (Figure 2). Specifically, the synthesis of these compounds, as previously described [22], was achieved following the build/couple/pair approach by applying different synthetic strategies consisting of no more than four/five steps.

**Figure 3:**
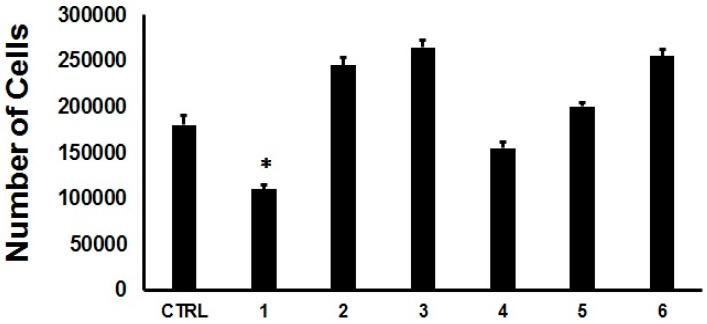
MDA-MB-231 cell number after 48 h of incubation with the selected molecules at 10 µM concentration. Values correspond to the mean of three independent experiments. Error bars indicate the corresponding standard deviations values. Student’s *t* test was used to evaluate the data significance, * *p* < 0.05.

A first screening on MDA-MB-231 human breast carcinoma cells was performed for the six compounds at 10 μM concentration. After the first 24 h of treatment, no particular effects on cell growth were observed (data not shown). Interestingly, after 48 h incubation, although a slightly induction in cell proliferation was detected for compounds, **2**, **3** and **6**, we observed a significant reduction in cell proliferation following the treatment with compound **1**, containing the hexahydro-2*H*-furo[3,2-*b*][1,4]oxazine scaffold (*p* < 0.05), with a 40% inhibition of cell growth (Figure 3). A similar range of inhibition was found by others [54].

**Figure 4:**
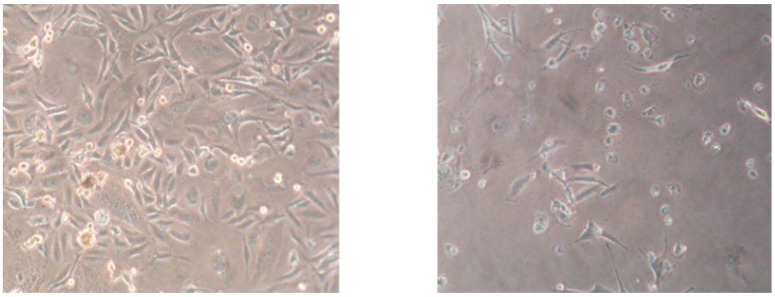
Control cells after 48 h (**left**), and cells after 48 h of incubation with compound **1** at 10 µM concentration (**right**).

**Figure 5:**
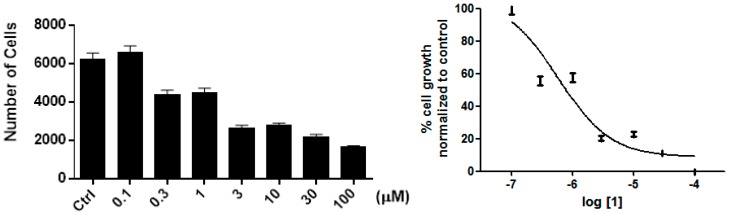
**Left**: Cell growth inhibition in MDA-MB-231 cells after 48 h of incubation with compound **1** at the reported concentration; **Right**: dose-response curve of log[**1**] vs. % cell growth normalized to control. Values indicate the mean of three independent experiments. Error bars show the corresponding standard deviation values.

Examination of cell morphology reveals essential information regarding the healthy status of a cell population. This inspection indicated that, after 48 h incubation, MDA-MB-231 cells were reduced in number, and part of the cell population showed a regular appearance, while part exhibited a round shape, though still adhering to the substrate. This observation suggests that the effect of compound **1** might induce a cell cycle slowdown and might not be related to any perturbation of the adhesive properties of the cell (Figure 4). In agreement with this, we found a dose-dependent cell growth inhibition, and we observed a significant reduction in cell proliferation at a concentration higher than 3 µM (Figure 5), with an IC_50_ of 0.6 μM.

**Scheme 1:**
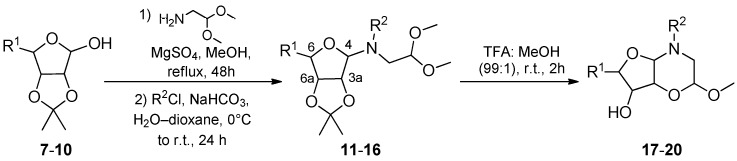
Two step synthesis of hexahydro-2*H*-furo[3,2-*b*][1,4]oxazine compounds.

In order to study and improve the activity of this compound, a pool of hexahydro-2*H*-furo[3,2-*b*][1,4]oxazine compounds with different polyhydroxylated chains, stereochemistry, and amine functionalities, were synthetized following the two-step process as reported in Scheme 1 and Table 1.

The reaction of the sugar derivatives **7**–**10** with glycine-derived amino acetaldehyde by formation of the glycosyl amine and the direct *N*-acylation of the crude hemiaminal coupling intermediates resulted in the achievement of compounds **11**–**16**. Then, under acid-catalyzed *trans*-acetalization conditions, the reaction of the *C*-3a hydroxyl group of the intermediate with the dimethylacetal carbon atom, led to the synthesis of the corresponding bicyclic compounds **17**–**20**. In this way, as reported in Table 1, the compounds with different polyhydroxylated chains and different stereochemistry were obtained starting from the appropriate furanosidic monosaccharide. Specifically, from 2,3-*O*-isopropylidene-d-ribofuranose (**7**) and 2,3-*O*-isopropylidene-d-lyxofuranose (**8**) the corresponding Fmoc-protected intermediates **11α**/**11β** and **12α**/**12β** were obtained with a clean conversion and a successful separation of the two anomers, similarly to what obtained from 2,3:5,6-*O*-di-isopropylidene-d-mannose **10**, as reported [22]. The assignment of the configuration of the anomers was established by analysing the coupling constants between the protons of the CH_2_N-moiety of the corresponding deprotected mannose-derived coupling intermediates, which are more stable and achievable by column chromatography, according to data reported in the literature for similar amino-α-d-mannofuranoses [55].

The preferred anomer of compounds **11**–**16** were found to be the α-anomer, even though starting from lyxose the ratio between the two anomeric compounds **12** became approximately 1:1 (Table 1, entry 2). The instability of the erythrose-derived coupling intermediate was evinced by the low yield of the reaction and in the achievement of only the most stable α-anomer product **13α** (Table 1, entry 3).

Upon treating both the anomers of the coupling intermediates under neat TFA conditions, the corresponding bicyclic *cis*-fused scaffolds **17**–**20** were obtained, even if in less yield as compared to the mannose-derived bicycle **1** (entry 4). In all cases, the same scaffold was achieved from both the anomers in similar yields as a consequence of a thermodynamic equilibration of *N*-Fmoc hemiaminal intermediate species under the acidic treatment [56].

**Scheme 2:**
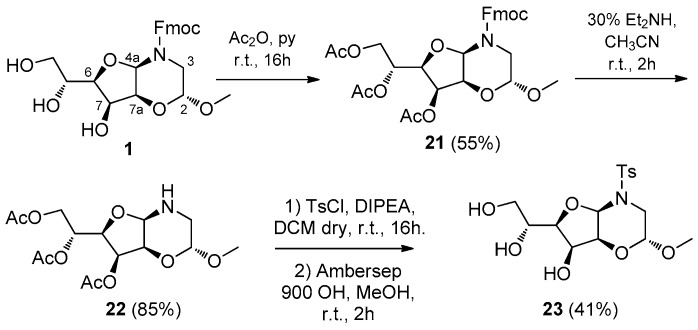
Functionalization of the hemiaminal function of hexahydro-2*H*-furo[3,2-*b*][1,4]oxazine **1**.

**Figure 6:**
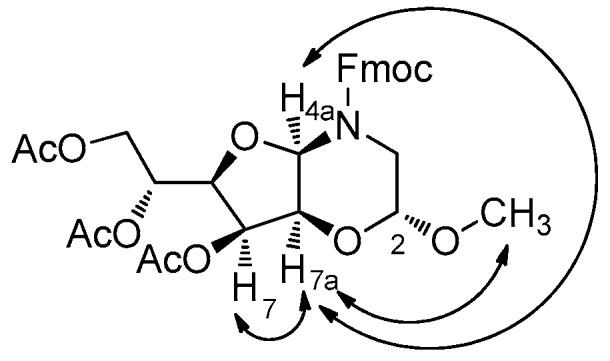
Selected nOe contacts from NOESY1D spectra of **21**.

The *cis*-fusion was evinced by NOESY1D experiments of the fully acetylated compound **21** (Scheme 2 and Figure 6). The same *cis*-fusion was evinced for the other scaffolds thanks to the diagnostic signal of the bridgehead protons which appeared as singlets in a diagnostic and unambiguous region of ^1^H-NMR spectrum between 5.70 and 4.70 ppm. In all cases only the *endo* anomer was recovered, which is the most stable for stereoelectronic effects.

The derivatization of the unstable hemiaminal coupling intermediate was possible only using benzoyl chloride (entry 5) and chloroformates (entries 4 and 6). However, even if the use of chloroformate resulted in a clean cyclization to the final product, benzoyl analogues (**15α**/**15β**) failed to give the corresponding bicyclic compound, as a consequence of the reduced stability of the *N*-acyl moiety.

The reaction of the hemiaminal intermediate with sulfonyl chloride was not possible at all. However, in order to explore the possibility of introducing a different moiety linked to the amine and studying their biological effect, the tosyl derivative **23** was obtained from the corresponding per-*O*-acetylated *N*-deprotected **22** (Scheme 2) subsequent to Fmoc deprotection of **21**.

In order to assess the stability of this class of bicyclic compounds based on the hexahydro-2*H*-furo[3,2-*b*][1,4]oxazine scaffold concerning the hemiaminal moiety under acidic conditions, a study by HPLC analysis was performed on **18** as a representative compound in MeOH and acetonitrile as solvents and in the presence of 1 M HCl, after 1 h and 24 h. In all cases, neither the formation of by-products nor the reduction of peak intensity was evinced, as compared to benzophenone as an internal standard (see Supplementary Materials for HPLC data).

These new compounds were tested for their inhibition activity at 10 µM concentration after 48 h of incubation on the same MDA-MB-231 cell line. As reported in Table 2, none of these compounds proved to be as interesting as **1**, revealing a specific structural requirement for an optimal inhibition profile. The importance of the number hydroxyl groups and their stereochemistry for the activity was evinced by comparing the inhibition activities of Fmoc-derivatives **1** and **17**–**19**, and those of **1** and the corresponding fully acetylated derivative **21**. Finally, an indication of the importance of the functionalization of the hemiaminal nitrogen atom was shown by comparing the inhibition activities of **1**, **20**, and **23**, all possessing similar scaffold and polyhydroxylated tail, and differing by the Fmoc, Cbz and tosyl group at the nitrogen atom, respectively. All in all, the biological data suggested a specific effect of the bicyclic compounds towards cell growth inhibition of MDA-MB-231 cells, as any small change in the structure or appendage of the lead compound **1** caused a drop in the biological activity.

**Figure 7:**
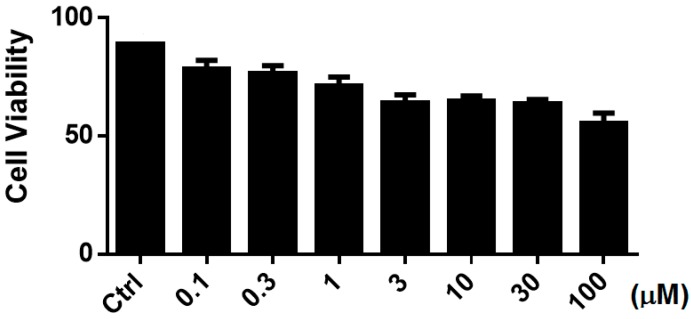
Cell viability assay (WST-1) in MDA-MB-231 cells after 48 h of incubation with compound **1** at the reported concentration. Values indicate the mean of three independent experiments and are expressed as percentage compared to control cells, error bars indicate the corresponding standard deviations values.

**Figure 8:**
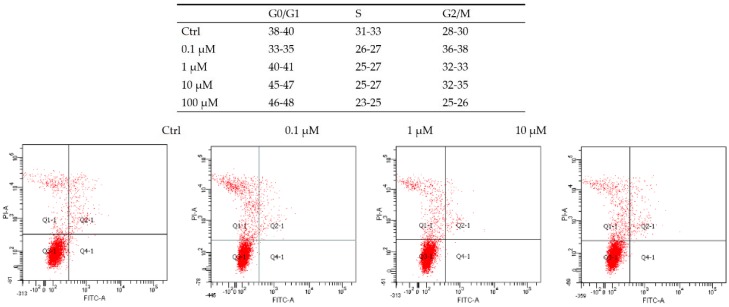
Cell cycle assay (upper panel) in MDA-MB-231 cells after 48 h of incubation with compound **1** at the reported concentration. Values indicate the range of cell population percentage in different cell cycle phases of three independent experiments. Apoptosis assay (lower panel) in MDA-MB-231 cells after 48h of incubation with compound **1** at the reported concentration. Representative cell plot analysis of apoptosis in MDA-MB-231.

We investigated the effect of compound **1** in cell viability using the WST-1 tetrazolium salt assay. The stable tetrazolium salt WST-1 is cleaved to a soluble formazan by a mechanism that is largely dependent on the glycolytic production of NAD(P)H in viable cells, thus, the amount of formazan dye produced directly correlates with the number of metabolically active cells. We tested different concentrations of compound **1**, and we found that the number of dead cell was not significantly different as compared to the control (Figure 7). Moreover, we investigated the effect of compound **1** on MDA-MB-231 cell cycle. We found that, starting from 10 μM concentration, this compound clearly induced a significant arrest of MDA-MB-231 cell cycle (Figure 8, upper panel). The effect on cell apoptosis was evaluated in the entire exposed cell population. As mentioned before, no significative effect on cell adhesion was found, however, after the 48 h treatment, the very few cells present in the supernatant and the adherent cells were grouped together and no differences were observed between the diverse cell treatments in terms of apoptosis (Figure 8, lower panel). Overall, it is possible to assume that the growth inhibition induced by compound **1** on MDA-MB-231 cells, might be correlated to a cytostatic effect, rather than to a cytotoxic one. Thus, the exploration of the fine mechanisms that regulate the cell cycle will be performed in future to identify the protein target of compound **1**, and future experiments on non-transformed epithelial cells will be performed to ensure the selectivity and specificity of the compounds [57].

The identification of the target responsible for the observed phenotype, above the many different growth factor signals and cyclin-dependent kinases will shed light in the understanding of tumor cell growth and progression [58,59]. Moreover, further investigations will be attempted in view to characterize the signaling pathways underlying the biological effect [60,61,62], particularly investigating the role of caspases and the modulation of their phosphorylation [60,63,64].

## 3. Materials and Methods

### 3.1. General Methods

Analytical grade solvents and commercially available reagents were used without further purification. Flash column chromatography (FCC) purifications were performed manually using glass columns with Merck silica gel (0.040–0.063 mm, Merck, Darmstad, Germany), or using the Isolera system (Biotage, Uppsala, Sweden) and SNAP silica cartridges. TLC analyses were performed on Merck silica gel 60 F254 plates. ^1^H-NMR and ^13^C-NMR spectra were recorded on a Mercury 400 (^1^H: 400 MHz, ^13^C: 100 MHz), or a Gemini 200 (^1^H: 200 MHz, ^13^C: 50 MHz) instrument (Varian, Palo Alto, CA, USA). HSQC experiments were carried out to define the multiplicity of ^13^C signals. All chemical shifts are reported in parts per million (δ) referenced to residual nondeuterated solvent. Data are reported as follows: chemical shifts, multiplicity (br = broad, s = singlet, d = doublet, t = triplet, q = quartet, m = multiplet; coupling constant(s) in Hz; integration). ESI mass spectra were carried out on a ion-trap double quadrupole mass spectrometer using electrospray (ES+) ionization techniques, and a normalized collision energy within the range of 25−32 eV for MSMS experiments. IR spectra were recorded with a FTIR-1600 spectrophotometer (Perkin-Elmer, Waltham, MA, USA). Elemental analyses were performed on a Perkin Elmer 240 C, H, N analyzer. Optical rotation measurements were performed on a DIP-370 polarimeter (JASCO Easton, MD, USA) and are given in 10^−1^ deg·cm^2^·g^−1^.

### 3.2. (9H-Fluoren-9-yl)methyl (2,2-dimethoxyethyl)((3aR,4S,6R,6aR)-6-(hydroxymethyl)-2,2-dimethyl-tetrahydrofuro[3,4-d][1,3]dioxol-4-yl)carbamate *(**11α**)* and (9H-Fluoren-9-yl)-methyl-(2,2-dimethoxyethyl)-((3aR,4R,6R,6aR)-6-(hydroxymethyl)-2,2-dimethyltetrahydrofuro[3,4-d][1,3]dioxol-4-yl)carbamate *(**11β**)*

To a solution of 2,3-*O*-isopropylidene-d-ribofuranose (**7**, 800 mg, 4.20 mmol) [65] and 2,2-dimethoxyethylamine (0.55 mL, 5.04 mmol) in MeOH (24 mL), MgSO_4_ (1.010 g, 8.40 mmol) was added and the reaction mixture was left stirring at reflux for 48 h. MgSO_4_ was then removed by filtration through Celite and the filtrate was concentrated under vacuum to give the crude hemiaminal intermediate, which was unstable under silica gel column chromatography conditions. The crude compound was then dissolved in dioxane (5 mL) and in a solution of NaHCO_3_ (705 mg, 8.40 mmol) in water (10 mL). The mixture was cooled to 0 °C, then a solution of Fmoc-Cl (1.090 g, 8.40 mmol) in dioxane (5 mL) was added slowly and the resulting suspension was left reacting at room temperature for 24 h under a nitrogen atmosphere, then it was diluted with EtOAc (30 mL). The organic phase was washed with 1M HCl solution, brine, and dried over anhydrous Na_2_SO_4_. After solvent evaporation, the crude oil was purified by flash chromatography (EtOAc/Pet. ether = 1:2; *R*_f_ **11β** = 0.31, *R*_f_ **11α** = 0.14), thus affording compound **11β** (418 mg, 0.84 mmol, 20%) and compound **11α** (796 mg, 1.59 mmol, 38%), both as colorless oils.

**11α**: ${\left[\alpha \right]}_{D}^{20}$ = −50.6 (*c* 1.4, CHCl_3_). ^1^H-NMR (400 MHz, CDCl_3_) δ 7.68 (d, *J* = 7.4 Hz, 2H), 7.64–7.57 (m, 2H), 7.32 (pt, *J* = 7.3 Hz, 2H), 7.29 (pt, *J* = 7.4 Hz, 2H), 5.77 (br s, 0.5H), 5.20 (br s, 0.5H), 4.82–4.41 (m, 5H), 4.27 (pt, *J* = 5.2 Hz, 1H), 4.18 (br s, 0.5H), 3.85 (br s, 0.5H), 3.69–3.54 (m, 3H), 3.43–3.37 (m, 1H), 3.20 (s, 6H), 2.05 (br s, 1H, OH), 1.41–1.38 (m, 3H), 1.24–1.18 (m, 3H). ^13^C-NMR (100 MHz, CDCl_3_) mixture of rotamers: δ 156.0 (s), 143.9 (s, 2C), 141.3 (s, 2C), 127.6 (d, 2C), 127.1 (d, 2C), 124.9 (d, 2C), 119.8 (d, 2C), 112.7 (s), 103.4 e 102.5 (d), 95.0 (d), 86.2 (d), 82.6 (d), 80.0 (d), 67.2 and 67.0 (t), 63.2 (t), 53.4 (q, 2C), 47.2 (d), 45.1 and 45.0 (t), 26.0 (q), 24.1 (q). MS (ESI) *m*/*z* (%): 521.60 [(M + Na)^+^, 100]. IR (CDCl_3_): ν = 3608, 2940, 1701, 1452, 1384, 1215 cm^−1^. Anal. Calcd. for C_27_H_33_NO_8_: C, 64.92; H, 6.66; N, 2.80. Found: C, 65.22; H, 6.71; N, 2.63.

**11β**: ${\left[\alpha \right]}_{D}^{19}$ = −12.2 (*c* 1.3, CHCl_3_). ^1^H-NMR (400 MHz, CDCl_3_) mixture of rotamers: δ 7.75 (d, *J* = 7.5 Hz, 2H), 7.58 (d, *J* = 7.6 Hz, 2H), 7.38 (pt, *J* = 7.0 Hz, 2H), 7.31 (pt, *J* = 7.4 Hz, 2H), 4.79 (br s, 1.5H), 4.60 (d, *J* = 7.4 Hz, 2H), 4.38 (s, 0.5H), 4.21 (pt, *J* = 5.2 Hz, 1H), 4.02 (br s, 2H), 3.81 (d, *J* = 12.7 Hz, 1H), 3.66 (d, *J* = 7.4 Hz, 1H), 3.45–3.08 (m, 4H), 3.23 (s, 6H), 1.51 (s, 3H), 1.30 (s, 3H). ^13^C-NMR (100 MHz, CDCl_3_) mixture of rotamers: δ 155.4 (s), 143.6 (s, 2C), 141.4 and 141.3 (s, 2C), 127.8 (d, 2C), 127.2 and 127.1 (d, 2C), 124.8 (d, 2C), 120.0 (d, 2C), 112.5 (s), 103.6 (d), 96.5 (d), 88.0 (d), 85.5 (d), 79.8 (d), 67.8 (t), 63.9 (t), 56.9 and 55.2 (q, 2C), 50.7 (t), 47.2 (d), 27.3 (q), 25.4 (q). MS (ESI) *m*/*z* (%): 521.88 [(M + Na)^+^, 100]. IR (CDCl_3_): ν = 3590, 2939, 1706, 1472, 1386, 1261 cm^−1^. Anal. Calcd. for C_27_H_33_NO_8_: C, 64.92; H, 6.66; N, 2.80. Found: C, 65.26; H, 6.74; N, 2.61.

### 3.3. (9H-Fluoren-9-yl)methyl (2,2-dimethoxyethyl)((3aS,4S,6R,6aS)-6-(hydroxymethyl)-2,2-dimethyltetra-hydrofuro[3,4-d][1,3]dioxol-4-yl)carbamate *(**12α**)* and (9H-Fluoren-9-yl)-methyl-(2,2-dimethoxyethyl)-((3aS,4R,6R,6aS)-6-(hydroxymethyl)-2,2-dimethyltetrahydrofuro[3,4-d][1,3]dioxol-4-yl)carbamate *(**12β**)*

Compounds **12α**/**12β** were synthesized from 2,3-*O*-isopropylidene-d-lyxofuranose **8** [66,67] (826 mg, 4.34 mmol) as reported for **11α**/**11β**. After solvent evaporation, the crude oil was purified by flash chromatography (EtOAc/Pet. ether = 1:1; *R*_f_ **12β** = 0.52; *R*_f_ **12α** = 0.17), thus affording compound **12β** (776 mg, 1.55 mmol, 31%) and compound **12α** (816 mg, 1.63 mmol, 32%), both as white foam.

**12α**: ${\left(\alpha \right]}_{D}^{21}$ = +50.4 (*c* 1.3, CHCl_3_). ^1^H-NMR (400 MHz, CDCl_3_) mixture of rotamers δ 7.68 (d, *J* = 7.2 Hz, 2H), 7.59–7.41 (m, 2H), 7.32 (pt, *J* = 7.2 Hz, 2H), 7.26–7.22 (m, 2H), 5.56 (br s, 0.5H), 5.31 (br s, 0.5H), 4.97 (br s, 0.5H), 4.70–4.37 (m, 4.5H), 4.19 (pt, *J* = 5.2 Hz, 1H), 3.90–3.63 (m, 2H), 3.46–3.40 (m, 1H), 3.38 (s, 6H), 3.25–3.19 (m, 2H), 2.15 (br s, 1H, OH), 1.36–1.13 (m, 6H). ^13^C-NMR (100 MHz, CDCl_3_) mixture of rotamers: δ 155.7 (s), 143.9 (s, 2C), 141.3 (s, 2C), 127.8 and 127.7 (d, 2C), 127.2 and 127.1 (d, 2C), 125.0 and 124.6 (d, 2C), 120.0 and 119.8 (d, 2C), 112.7 (s), 103.4 e 102.6 (d), 86.8 (d), 85.0 (d), 79.9 and 79.5 (d), 78.2 (d), 68.1 and 67.5 (t), 60.6 (t), 52.5 (q), 50.7 (q), 47.2 (d), 45.5 (t), 25.5 and 25.1 (q), 23.9 and 23.7 (q). MS (ESI) *m*/*z* (%): 522.28 [(M + Na)^+^, 100]. IR (CDCl_3_): ν = 3075, 2939, 1706, 1261 cm^−1^. Anal. Calcd. for C_27_H_33_NO_8_: C, 64.92; H, 6.66; N, 2.80. Found: C, 65.23; H, 6.70; N, 2.64.

**12β**: ${\left(\alpha \right]}_{D}^{22}$ = +4.1 (*c* 1.3, CHCl_3_). ^1^H-NMR (400 MHz, CDCl_3_) mixture of rotamers: δ 7.76 (d, *J* = 7.4 Hz, 2H), 7.59 (d, *J* = 7.2 Hz, 2H), 7.40 (pt, *J* = 7.3 Hz, 2H), 7.33 (pt, *J* = 7.3 Hz, 2H), 5.06–4.80 (m, 4H), 4.60–4.45 (m, 3H), 4.21 (pt, *J* = 5.2 Hz, 1H), 4.05–4.02 (m, 1H), 3.90–3.78 (m, 3H), 3.26 (s, 6H), 2.06 (br s, 1H, OH), 1.46 (m, 3H), 1.30 (s, 3H). ^13^C-NMR (100 MHz, CDCl_3_) mixture of rotamers: δ 155.8 (s), 143.6 (s, 2C), 141.4 and 141.3 (s, 2C), 127.8 (d, 2C), 127.2 (d, 2C), 124.7 (d, 2C), 120.0 (d, 2C), 112.4 (s), 103.5 (d), 96.4 (d), 85.8 (d), 83.6 (d), 82.4 (d), 66.7 (t), 62.0 (t), 55.2 (q, 2C), 50.4 (t), 47.3 (d), 26.2 and 25.9 (q), 24.3 (q). MS (ESI) *m*/*z* (%): 522.28 [(M + Na)^+^, 100]. IR (CDCl3): ν = 3567, 2901, 1703, 1448, 1354, 1216 cm^−1^. Anal. Calcd. for C_27_H_33_NO_8_: C, 64.92; H, 6.66; N, 2.80. Found: C, 65.26; H, 6.78; N, 2.60.

### 3.4. (9H-Fluoren-9-yl)methyl (2,2-dimethoxyethyl)((3aS,4S,6aS)-2,2-dimethyltetrahydrofuro[3,4-d][1,3]-dioxol-4-yl)carbamate *(**13α**)*

Compound **13α** was obtained from 2,3-*O*-isopropylidene-l-erythrofuranose (**9**, 920 mg, 5.74 mmol) as a single product with the same procedure reported for **11α**/**11β**. After solvent evaporation, the crude oil was purified by flash chromatography (EtOAc/Petr. et. = 1:3; *R*_f_ **13α** = 0.29), thus affording the α-anomer **13α** (834 mg, 1.78 mmol, 32%) as a colourless oil. Anomer β was recovered only in traces (<10 mg). ${\left(\alpha \right]}_{D}^{22}$ = +72.9 (*c* 1.4, CHCl_3_). ^1^H-NMR (400 MHz, CDCl_3_) mixture of rotamers: δ 7.76 (d, *J* = 7.5 Hz, 2H), 7.69–7.58 (m, 2H), 7.40 (pt, *J* = 7.4 Hz, 2H), 7.34–7.29 (m, 2H), 5.32 (br s, 1H), 4.73–4.39 (m, 5H), 4.28 (t, *J* = 5.9 Hz, 1H), 4.10–3.99 (m, 1.5H), 3.81 (br s, 0.5H), 3.56 (br s, 2H), 3.30 (s, 6H), 1.47–1.24 (m, 6H). ^13^C-NMR (100 MHz, CDCl_3_) mixture of rotamers: δ 155.8 (s), 144.0 and 143.9 (s, 2C), 141.3 (s, 2C), 127.7 and 127.6 (d, 2C), 127.2 and 127.1 (d, 2C), 124.9 (d, 2C), 119.8 (d, 2C), 112.4 (s), 103.5 (d), 87.8 (d), 79.3 (d), 78.8 (d), 69.7 (t), 67.2 (t), 53.5 (q, 2C), 47.2 (d), 45.4 (t), 25.8 (q), 24.1 (q). MS (ESI) *m*/*z* (%): 492.36 [(M + Na)^+^, 100]. IR (CDCl_3_): ν = 2939, 1703, 1451, 1216 cm^−1^. Anal. Calcd. for C_26_H_31_NO_7_: C, 66.51; H, 6.65; N, 2.98. Found: C, 66.80; H, 6.71; N, 2.89.

### 3.5. N-(2,2-Dimethoxyethyl)-N-((3aS,4S,6R,6aS)-6-((R)-2,2-dimethyl-1,3-dioxolan-4-yl)-2,2-dimethyltetra-hydrofuro[3,4-d][1,3]dioxol-4-yl)benzamide *(**15α**)* and N-(2,2-Dimethoxyethyl)-N-((3aS,4R,6R,6aS)-6-((R)-2,2-dimethyl-1,3-dioxolan-4-yl)-2,2-dimethyltetrahydrofuro[3,4-d][1,3]dioxol-4-yl)benzamide *(**15β**)*

To the crude hemiaminal coupling intermediate, prepared from 2,3:5,6-*O*-di-isopropylidene-d-mannose **10** (60 mg, 0.23 mmol) as reported for **11α**/**11β**, and Et_3_N (70 µL, 0.51 mmol) in dry THF (0.5 mL), a solution of benzoyl chloride (31 µL, 0.27 mmol) in dry THF (0.5 mL) was added dropwise at 0°C. The mixture was allowed to reach room temperature and was left stirring for two days under a nitrogen atmosphere. Successively the mixture was washed with a saturated solution of NaHCO_3_, a solution of 1N HCl and brine. After solvent evaporation, the crude oil was purified by flash chromatography (EtOAc/Pet. ether = 1:1; *R*_f_ **15β** = 0.61, *R*_f_ **15α** = 0.39), thus affording compound **15β** (35 mg, 0.08 mmol, 33%) and compound **15α** (50 mg, 0.11 mmol, 49%), both as colorless oils.

**15α**: ${\left(\alpha \right]}_{D}^{23}$ = +63.0 (*c* 1.0, CHCl_3_). ^1^H-NMR (400 MHz, CDCl_3_) mixture of rotamers: δ 7.48–7.38 (m, 5H), 4.71 (br s, 2H), 4.43 (q, *J* = 5.8 Hz, 1H), 4.13–4.09 (m, 3H), 3.66–3.61 (m, 3H), 3.43 (s, 1H), 3.28 (br s, 3H), 1.52 (s, 3H), 1.44 (s, 3H), 1.38 (s, 3H), 1.32 (s, 3H). ^13^C-NMR (100 MHz, CDCl_3_) mixture of rotamers: δ 172.1 (s), 136.0 and 132.8 (s), 131.2 and 129.6 (d), 128.2 and 128.0 (d, 2C), 126.8 and 126.6 (d, 2C), 112.5 (s), 108.9 (s), 103.4 and 102.4 (d), 87.2 and 79.4 (d), 78.8 (d), 78.2 (d), 77.4 (d), 72.7 (d), 66.3 (t), 54.3 and 53.6 (q), 53.5 and 53.4 (q), 47.2 and 41.2 (t), 26.6 (q), 25.4 (q), 24.9 (q), 23.7 (q). MS (ESI) *m*/*z* (%): 474.20 [(M + Na)^+^, 100], 924.59 [(2M + Na)^+^, 16]. IR (CDCl_3_): ν = 3019, 1626, 1521, 1423, 1216, 1046 cm^−1^. Anal. Calcd. for C_23_H_33_NO_8_: C, 61.18; H, 7.37; N, 3.10. Found: C, 61.43; H, 7.45; N, 3.00.

**15β**: ${\left(\alpha \right]}_{D}^{23}$ = +8.0 (*c* 0.7, CHCl_3_). ^1^H-NMR (400 MHz, CDCl_3_) mixture of rotamers: δ 7.41–7.36 (m, 5H), 5.20 (d, *J* = 7.0 Hz, 2H), 5.01 (br s, 1H), 4.54 (br s, 2H), 4.32 (q, *J* = 5.9 Hz, 1H), 4.06 (dd, *J* = 8.1, 6.3 Hz, 1H), 3.99 (dd, *J* = 8.1, 5.2 Hz, 1H), 3.57 (dd, *J* = 14.6, 5.6, 1H), 3.38 (s, 3H), 3.31 (s, 3H), 3.38–3.22 (m, 1H), 1.43 (s, 6H), 1.36 (s, 3H), 1.32 (s, 3H). ^13^C-NMR (100 MHz, CDCl_3_) major rotamer: δ 168.6 (s), 135.8 (s), 130.1 (d), 128.4 (d, 2C), 127.4 (d, 2C), 108.9 (s), 103.8 (s), 95.0 (d), 85.7 (d), 85.1 (d), 84.9 (d), 81.5 (d), 74.0 (d), 66.5 (t), 56.1 (q, 2C), 48.4 (t), 26.9 (q, 2C), 26.2 (q), 25.2 (q). MS (ESI) *m*/*z* (%): 474.19 [(M + Na)^+^, 100]. IR (CDCl_3_): ν = 3016, 1625, 1520, 1427, 1220, 1041 cm^−1^. Anal. Calcd. for C_23_H_33_NO_8_: C, 61.18; H, 7.37; N, 3.10. Found: C, 61.46; H, 7.49; N, 2.98.

### 3.6. Benzyl (2,2-dimethoxyethyl)((3aS,4R,6R,6aS)-6-((R)-2,2-dimethyl-1,3-dioxolan-4-yl)-2,2-dimethyl-tetrahydrofuro[3,4-d][1,3]dioxol-4-yl)carbamate *(**16α**)* and Benzyl (2,2-dimethoxyethyl)((3aS,4S,6R,6aS)-6-((R)-2,2-dimethyl-1,3-dioxolan-4-yl)-2,2-dimethyltetrahydrofuro[3,4-d][1,3]dioxol-4-yl)carbamate *(**16β**)*

The crude hemiaminal coupling intermediate, prepared from 2,3:5,6-*O*-di-isopropylidene-d-mannose (**10**, 107 mg, 0.41 mmol) as reported for **11α**/**11β**, and NaHCO_3_ (70 mg, 0.82 mmol) were dissolved in a mixture of H_2_O (4 mL) and EtOAc (2 mL), then Cbz-Cl (68 µL, 0.48 mmol) in EtOAc (2 mL) was added dropwise at 0 °C. The mixture was allowed to reach room temperature and was left stirring overnight under a nitrogen atmosphere. Successively, the mixture was washed with aqueous 1N HCl and brine. The organic phase was dried over anhydrous Na_2_SO_4_ and concentrated under reduced pressure. After solvent evaporation, the crude oil was purified by flash chromatography (EtOAc/Pet. ether = 1:3; *R*_f_ **16β** = 0.55, *R*_f_ **16α** = 0.42), thus affording compound **16β** (43 mg, 0.09 mmol, 22%) and compound **16α** (99 mg, 0.21 mmol, 50%), both as colorless oils.

**16α**: ${\left(\alpha \right]}_{D}^{23}$ = +45.9 (*c* 0.9, CHCl_3_). ^1^H-NMR (400 MHz, CDCl_3_) mixture of rotamers: δ 7.35 (s, 5H), 5.11 (br s, 5H), 4.69 (s, 0.5H), 4.42–4.31 (m, 2.5H), 4.07–4.00 (m, 1H), 3.89 (br s, 0.5H), 3.80–3.78 (m, 0.5H), 3.69 (br s, 1H), 3.36–3.33 (m, 7H), 1.49–1.43 (m, 6H), 1.36–1.32 (m, 6H). ^13^C-NMR (50 MHz, CDCl_3_) mixture of rotamers: δ 155.7 (s), 135.9 (s), 129.0 and 128.9 (d), 128.8 and 128.6 (d, 2C), 128.2 and 128.1 (d, 2C), 112.6 and 112.4 (s), 108.9 (s), 103.8 and 103.7 (d), 96.7 and 96.6 (d), 85.8 (d), 84.3 (d), 82.1 and 81.5 (d), 74.0 and 71.2 (d), 67.9 and 66.6 (t), 64.6 (t), 55.1 and 55.0 (q, 2C), 50.6 (t), 26.8 (q), 26.2 (q), 25.3 (q), 24.4 (q). MS (ESI) *m*/*z* (%): 504.08 [(M + Na)^+^, 100]. IR (CDCl_3_): ν = 3453, 2940, 1709, 1383, 1211 cm^−1^. Anal. Calcd. for C_24_H_35_NO_9_: C, 59.86; H, 7.33; N, 2.91. Found: C, 60.12; H, 7.41; N, 2.84.

**16β**: ${\left(\alpha \right]}_{D}^{23}$ = +8.0 (*c* 0.8, CHCl_3_). ^1^H-NMR (400 MHz, CDCl_3_) mixture of rotamers: δ 7.31–7.29 (m, 5H), 5.61 and 5.45 (s, 1H), 5.14–5.07 (m, 2H), 4.71–4.64 (m, 2H), 4.39–4.29 (m, 2.5H), 4.06–3.78 (m, 3H), 3.61–3.42 (m, 1.5H), 3.30–3.18 (m, 3H), 3.23 (s, 3H), 1.43–1.21 (m, 12H). ^13^C-NMR (50 MHz, CDCl_3_) mixture of rotamers: δ 155.2 (s), 135.7 (s), 130.5 (d), 128.6 and 128.4 (d, 2C), 128.1 and 127.2 (d, 2C), 113.3 (s), 109.3 and 108.4 (s), 85.5 and 85.4 (d), 79.9 (d), 79.7 and 79.6 (d), 78.4 (d), 72.9 (d), 69.9 and 69.8 (d), 68.2 (t), 66.9 and 66.8 (t), 64.3 (t), 52.7 and 52.6 (q), 52.5 (q), 30.8 and 29.7 (q), 28.4 (q), 26.9 (q), 26.3 and 25.1 (q). MS (ESI) *m*/*z* (%): 504.08 [(M + Na)^+^, 100]. IR (CDCl_3_): ν = 3456, 2985, 1703, 1261 cm^−1^. Anal. Calcd. for C_24_H_35_NO_9_: C, 59.86; H, 7.33; N, 2.91. Found: C, 60.16; H, 7.44; N, 2.81.

### 3.7. (2R,4aS,6R,7R,7aR)-(9H-Fluoren-9-yl)methyl 7-hydroxy-6-(hydroxymethyl)-2-methoxytetrahydro-2H-furo[3,2-b][1,4]oxazine-4(3H)-carboxylate *(**17**)*

Compound **11α** (134 mg, 0.27 mmol) was dissolved in trifluoroacetic acid (1 mL) and MeOH (100 µL) and stirred at room temperature for 2 h. After TFA evaporation, the crude powder was purified by flash chromatography (EtOAc/Pet. ether = 1:1; *R*_f_ **17** = 0.40), thus affording compound **17** as a white foam (62 mg, 0.15 mmol, 55%). ${\left(\alpha \right]}_{D}^{19}$ = +52.9 (*c* 0.09, CHCl_3_). ^1^H-NMR (400 MHz, CDCl_3_) mixture of rotamers: δ 7.69 (d, *J* = 7.5 Hz, 2H), 7.50 (pt, *J* = 6.9 Hz, 2H), 7.33 (pt, *J* = 7.5 Hz, 2H), 7.26–7.22 (m, 2H), 5.13 and 4.57 (s, 1H), 4.74 (d, *J* = 3.9 Hz, 1H), 4.77–4.74 and 4.62–4.57 (m, 1H), 4.41–4.36 (m, 1H), 4.20 (t, *J* = 7.4 Hz, 1H), 4.21–4.15 (m, 1H), 4.05–3.96 (m, 2H), 3.86–3.77 (m, 1H), 3.67–3.65 (br s, 1H), 3.59–3.46 (m, 1.5H), 3.38 and 3.32 (s, 3H), 3.41–3.27 (m, 0.5H), 2.31 (br s, 2H). ^13^C-NMR (50 MHz, CDCl_3_) mixture of rotamers: δ 155.4 (s), 143.9 and 143.7 (s, 2C), 141.4 (s, 2C), 127.8 (d, 2C), 127.1 (d, 2C), 125.0 (d, 2C), 120.0 (d, 2C), 95.6 and 95.5 (d), 78.8 (d), 73.0 (t), 69.0 (t), 68.5 and 68.4 (t), 67.7 (d), 66.6 (d), 55.0 and 54.9 (q), 47.1 (d), 42.4 and 42.3 (t). MS (ESI) *m*/*z* (%): 449.49 [(M + Na)^+^, 100]. IR (CDCl_3_): ν = 3608, 3157, 1474, 1382, 1216 cm^−1^. Anal. Calcd. for C_23_H_25_NO_7_: C, 64.63; H, 5.90; N, 3.28. Found: C, 64.80; H, 5.97; N, 3.21.

### 3.8. (2R,4aR,6R,7S,7aS)-(9H-Fluoren-9-yl)methyl 7-hydroxy-6-(hydroxymethyl)-2-methoxytetrahydro-2H-furo[3,2-b][1,4]oxazine-4(3H)-carboxylate *(**18**)*

Compound **12β** (250 mg, 0.50 mmol) was dissolved in trifluoroacetic acid (1 mL) and MeOH (100 µL) and stirred at room temperature for 2 h. After TFA evaporation, the crude powder was purified by flash chromatography (EtOAc/Pet. ether = 1:1; *R*_f_ **18** = 0.43), thus affording compound **18** as a white foam (142 mg, 0.33 mmol, 66%). With the same procedure, compound **18** (153 mg, 0.36 mmol, 68%) was obtained also from the diastereomer **12α** (264 mg, 0.53 mmol). ${\left(\alpha \right]}_{D}^{19}$ = −51.1 (*c* 1.2, CHCl_3_). ^1^H-NMR (400 MHz, CDCl_3_) mixture of rotamers: δ 7.70 (d, *J* = 7.5 Hz, 2H), 7.51 (pt, *J* = 6.6 Hz, 2H), 7.34 (pt, *J* = 7.3 Hz, 2H), 7.25 (pt, *J* = 6.9 Hz, 2H), 5.21 and 4.82 (s, 1H), 4.72 (d, *J* = 8.9 Hz, 1H), 4.59–4.56 (m, 1H), 4.42–4.13 (m, 2H), 3.99–3.76 (m, 3H), 3.54–3.39 (m, 2H), 3.38 and 3.32 (s, 3H), 3.26–3.19 (m, 1H), 3.02 (pt, *J* = 10.1 Hz, 1H), 2.24 (br s, 2H). ^13^C-NMR (100 MHz, CDCl_3_) mixture of rotamers: δ 156.0 (s), 143.8 (s, 2C), 141.3 (s, 2C), 127.8 (d, 2C), 127.1 (d, 2C), 125.0 (d, 2C), 120.0 (d, 2C), 95.0 (d), 78.8 (d), 73.9 (t), 68.3 (t), 67.7 (t), 66.6 (d), 66.0 (d), 55.0 (q), 47.1 and 46.7 (d), 42.6 and 41.5 (t). MS (ESI) *m*/*z* (%): 450.24 [(M + Na)^+^, 100], 876.66 [(2M + Na)^+^, 60]. IR (CDCl_3_): ν = 3610, 3155, 1472, 1382, 1216 cm^−1^. Anal. Calcd. for C_23_H_25_NO_7_: C, 64.63; H, 5.90; N, 3.28. Found: C, 64.87; H, 5.99; N, 3.22.

### 3.9. (2R,4aR,7S,7aS)-(9H-Fluoren-9-yl)methyl 7-hydroxy-2-methoxytetrahydro-2H-furo[3,2-b][1,4]oxazine-4(3H)-carboxylate *(**19**)*

Compound **13α** (203 mg, 0.43 mmol) was dissolved in trifluoroacetic acid (1 mL) and MeOH (100 µL) and stirred at room temperature for 2 h. After TFA evaporation, the crude powder was purified by flash chromatography (EtOAc/Pet. ether = 1:1; *R*_f_ **19** = 0.32), thus affording compound **19** as a white foam (80 mg, 0.20 mmol, 47%). ${\left(\alpha \right]}_{D}^{19}$ = −56.7 (*c* 1.0, CHCl_3_). ^1^H-NMR (400 MHz, CDCl_3_) mixture of rotamers: δ 7.68 (d, *J* = 7.5 Hz, 2H), 7.52–7.50 (m, 2H), 7.34–7.21 (m, 4H), 5.70 and 5.60 (s, 1H), 4.76 (d, *J* = 8.9 Hz, 1H), 4.48–4.18 (m, 4H), 4.07–4.01 (m, 2H), 3.68 (pt, *J* = 14.6 Hz, 1H), 3.72 (dd, *J* = 16.6 Hz, 8.6 Hz, 2H), 3.40 and 3.32 (s, 3H), 3.28–3.21 (m, 1H). ^13^C-NMR (100 MHz, CDCl_3_) mixture of rotamers: δ 155.8 (s), 143.8 and 143.6 (s, 2C), 141.3 and 141.2 (s, 2C), 127.7 (d, 2C), 127.1 (d, 2C), 125.2 and 125.0 (d, 2C), 119.9 (d, 2C), 95.3 and 94.9 (d), 81.2 and 80.8 (d), 71.8 (t), 71.6 (d), 68.2 (t), 66.5 and 66.1 (d), 55.3 and 54.5 (q), 47.0 (d), 42.2 and 41.7 (t). MS (ESI) *m*/*z* (%): 420.35 [(M + Na)^+^, 100]. IR (CDCl_3_): ν = 3619, 3020, 1714, 1451, 1221 cm^−1^. Anal. Calcd. for C_22_H_23_NO_6_: C, 66.49; H, 5.83; N, 3.52. Found: C, 67.01; H, 5.90; N, 3.40.

### 3.10. (2R,4aR,6R,7S,7aS)-Benzyl 6-((R)-1,2-dihydroxyethyl)-7-hydroxy-2-methoxytetrahydro-2H-furo[3,2-b][1,4]oxazine-4(3H)-carboxylate *(**20**)*

Compound **16α** (40 mg, 0.08 mmol) was dissolved in trifluoroacetic acid (1 mL) and stirred at room temperature for 2 h. After TFA evaporation, the crude powder was purified by flash chromatography (EtOAc, *R*_f_ **20** = 0.19), thus affording compound **20** as white foam (19 mg, 0.05 mmol, 65%). ${\left(\alpha \right]}_{D}^{23}$ = −51.6 (*c* 0.5, CHCl_3_). ^1^H-NMR (400 MHz, CDCl_3_) mixture of rotamers: δ 7.28 (s, 5H), 5.29 and 5.16 (s, 1H), 5.15–5.08 (m, 2H), 4.73 and 4.64 (s, 1H), 4.10 (s, 1H), 3.82–3.58 (m, 5H), 3.47–3.44 (m, 1H), 3.32 (s, 3H), 3.33–3.26 (m, 1.5H), 3.10–3.07 (m, 0.5H), 2.43 br s, 3H, OH). ^13^C-NMR (100 MHz, CDCl_3_) mixture of rotamers: δ 155.5 (s), 136.0 (s), 128.6 (d, 2C), 128.3 (d, 2C), 128.0 (d), 96.0 and 95.5 (d), 73.6 (d), 69.9 (d), 68.93 (d), 68.90 (d), 67.9 (t), 66.6 and 66.2 (t), 62.7 and 62.6 (d), 54.9 (q), 42.7 and 42.6 (t). MS (ESI) *m*/*z* (%): 392.05 [(M + Na)^+^, 100]. IR (CDCl_3_): ν = 3628, 3618, 3020, 1793, 1472, 1384, 1216 cm^−1^. Anal. Calcd. for C_17_H_23_NO_8_: C, 55.28; H, 6.28; N, 3.79. Found: C, 55.98; H, 6.40; N, 3.70.

### 3.11. (R)-1-((2R,4aR,6R,7S,7aS)-7-Hydroxy-2-methoxy-4-tosylhexahydro-2H-furo[3,2-b][1,4]oxazin-6-yl)ethane-1,2-diol *(**23**)*

To a solution of **22** [22] (45 mg, 0.14 mmol) and DIPEA (50 µL, 0.28 mmol) in dry CH_2_Cl_2_ (2 mL), a solution of TsCl (54 mg, 0.09 mmol) in dry CH_2_Cl_2_ (2 mL) was added slowly at 0 °C. The mixture was allowed to reach room temperature and was left stirring under a nitrogen atmosphere for 24 h. Then, water was added slowly and the resulting mixture was washed with a saturated solution of NaHCO_3_ and brine. The organic phase was dried over anhydrous Na_2_SO_4_ and concentrated under reduced pressure. The crude compound was left stirring in MeOH (2 mL) in presence of a catalytic amount of Ambersep 900 OH at room temperature for 1 h. The resin was then filtered and, after solvent evaporation, the crude mixture was purified by flash chromatography (EtOAc/Pet. ether = 1:1; *R*_f_ **23** = 0.19) thus affording pure compound **23** (23 mg, 0.06 mmol, 41%) as a white foam. ${\left(\alpha \right]}_{D}^{19}$ = −41.7 (*c* 0.6, CHCl_3_). ^1^H-NMR (400 MHz, CDCl_3_): δ 7.71 (d, *J* = 8.2 Hz, 2H), 7.25 (d, *J* = 8.2 Hz, 2H), 5.15 (s, 1H), 4.75 (s, 1H), 4.21 (d, *J* = 4.5 Hz, 1H), 3.72 (dd, *J* = 11.8, 3.5 Hz, 1H), 3.61 (dd, *J* = 9.7, 3.4 Hz, 1H), 3.56–3.50 (m, 3H), 3.39 (s, 3H), 3.31–3.27 (m, 1H), 3.08 (dd, *J* = 11.8, 2.6 Hz, 1H), 2.41 (s, 3H), 1.99 (br s, 3H, OH). ^13^C-NMR (50 MHz, CDCl_3_): δ 143.2 (s, 2C), 134.7 (s, 2C), 128.4 (d, 2C), 127.0 (d, 2C), 94.4 (d), 78.1 (d), 75.3 (d), 72.5 (d), 67.7 (d), 66.4 (d), 61.6 (t), 54.1 (q), 42.2 (t), 28.7 (q). MS (ESI) *m*/*z* (%): 412.23 [(M + Na)^+^, 100]. IR (CDCl_3_): ν = 3618, 3054, 1472, 1354, 1218, 1174 cm^−1^. Anal. Calcd. for C_16_H_23_NO_8_S: C, 49.35; H, 5.95; N, 3.60. Found: C, 49.91; H, 6.04; N, 3.48.

### 3.12. Cell Culture

Human MDA-MB-231 breast cancer cell lines are a model for aggressive, hormone-independent breast cancer. Cells were obtained from the American Tissue Culture Collection (Manassas, VA, USA) and maintained in DMEM (from Life Technologies, Carlsbad, CA, USA) containing 10% heat inactivated fetal calf serum (FCS, from Life Technologies) at 37 °C in a humidified atmosphere of 5% CO_2_ /95% air. Cells were harvested from subconfluent cultures by incubation with a trypsin–EDTA solution, and propagated every 3 days at a ratio between 1:4 and 1:8.

### 3.13. Cell Growth Assay

MDA-MB-231 cells were plated at 5 × 10^4^ cells/mL in 24-well plates under standard culture conditions. After 4h adhesion, cells were treated with either vehicle alone or different compounds. After 24 h or 48 h of treatments, cells were trypsinized, collected, and counted using a hemocytometer.

### 3.14. Cell Viability

Cell viability assay was performed using the WST-1 assay, a tetrazolium salt analog to MTT or XTT. Briefly, MDA-MB-231 cells were plated in in triplicate in a 96-well at 10–15 × 10^3^ cells/mL density. After 4 h adhesion, cells were treated with different concentration of compound **1**. Following 24 h or 48 h incubation, cells were exposed to 10 µL of the reagent directly to the cell cultures (200 µL final volume). The plates were incubated for 120 minutes at 37 °C in a humidified 5% CO_2_ environment. The WST-1 formazan product was measured at 460 nm (reference wavelength of 630 nm) with an ELx800 Universal Microplate Reader from Bio-Tek Instruments Inc. (Winooski, VT, USA).

### 3.15. Cell Cycle Analysis

MDA-MB231 cells were plated at 5 × 10^5^ cells/mL in 6-well plates under standard culture conditions. After 4 h adhesion, cells were treated with different concentration of compound **1**. After 48 h cells were harvested by trypsinization, and then permeabilized with 70% ice-cold ethanol on ice for 30 min. Cells were then washed and incubated in staining buffer with 50 μg/mL propidium iodide (PI), 10 μg/mL RNase A and 0.1% Triton X-100 for 30 min in the dark. Subsequently, the cell cycle was analyzed by flow cytometry (FACSCan; BD Biosciences, San Jose, CA, USA).

### 3.16. Data Analysis

The results are expressed as mean ± standard deviation (SD). Differences in growth rates between groups were analyzed using the two-tailed *t*-test, statistical significance at *p*-values <0.05 were presented using respective symbols in the figure legends.

## 4. Conclusions

In recent years, small molecules drug discovery and development became a highly challenging field, and in this view we have identified potential novel drug compounds starting from library compounds and investigated their effect on a malignant human tumor cell line. Specifically, the application of a cell-based growth inhibition assay on a library of skeletally different glycomimetics allowed for the selection of a novel chemotype based on the hexahydro-2*H*-furo[3,2-*b*][1,4]oxazine scaffold as a hit inhibitor of MDA-MB-231 cell growth. Subsequent follow-up synthesis of parent compounds and preliminary biological studies validated the selection of **1** as a valuable lead compound for the modulation of breast carcinoma cell cycle mechanism.

Although follow-up compounds showed reduced activity as compared to **1**, interesting structure-activity analysis showed a quite specific structural requirement, revealing that both the three hydroxyl group and the Fmoc group are crucial for inhibition. Compound **1** clearly induced a significant arrest of MDA-MB-231 cell cycle, and we assume that the growth inhibition induced by compound **1** on MDA-MB-231 cells might be correlated to a cytostatic effect, whereas no significative effect on cell adhesion and apoptosis was found.

Although we have found a cytostatic effect on tumor cells, future experiments on non-transformed epithelial cells will be performed to ensure the selectivity and specificity of the compounds [57]. The identification of the target responsible for the observed phenotype, above the many different growth factor signals and cyclin-dependent kinases will shed light in the understanding of tumor cell growth and progression [58,59]. Moreover, further investigations will be attempted in view to characterize the signaling pathways underlying the biological effect [60,61,62], particularly investigating the role of caspases and the modulation of their phosphorylation [60,63,64].

Further biological tests will be carried out starting from flow cytometry profiling and the use of reverse chemical genetics approaches to have insight into more detailed fine mechanisms that regulate the cell cycle [39,68,69].

## Figures and Tables

**Table 1:** Structures and yields of hexahydro-2*H*-furo[3,2-*b*][1,4]oxazine compounds.

Entry	Sugar Derivative	Coupling Intermediate	Hexahydro-2*H*-furo[3,2-*b*][1,4]oxazine
1	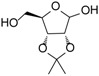 **7**	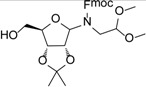 **11α**, 38%; **11β**, 20%	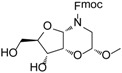 **17**, 55%
2	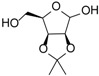 **8**	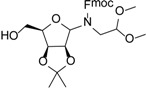 **12α**, 32%; **12β**, 31%	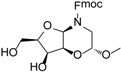 **18**, 68%
3	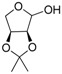 **9**	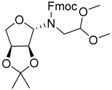 **13α**, 32%; **13β**, traces	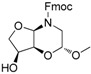 **19**, 47%
4	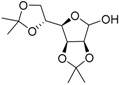 **10**	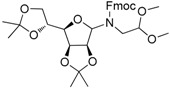 **14α**, 65%; **14β**, 22%	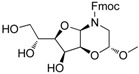 **1**, 76%
5	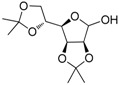 **10**	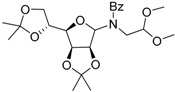 **15α**, 49%; **15β**, 33%	___
6	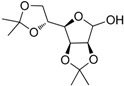 **10**	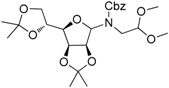 **16α**, 50%; **16β**, 22%	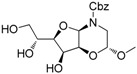 **20**, 65%

**Table 2:** Cell growth inhibition of MDA-MB-231 cell line at 10 µM concentration after 48 h of incubation.

Compoud	Structure	% Inhibition ^a^
**1**	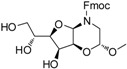	41 ± 3
**17**	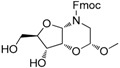	13 ± 1
**23**	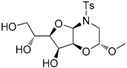	13 ± 3
**21**	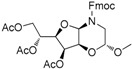	13 ± 2
**18**	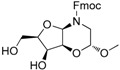	15 ± 11
**19**	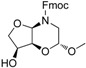	12 ± 6
**20**	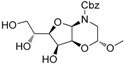	10 ± 4

^a^ Cell growth inhibition (% vs control) in MDA-MB-231 cells after 48 h of incubation with compound **1** at the reported concentration. Values ± SD indicate the mean of three independent experiments.

## Supplementary Materials

The following are available online at: http://www.mdpi.com/1420-3049/21/10/1405/s1, copies of ^1^H- and ^13^C-NMR spectra of **11**–**20** and **23**, and HPLC chromatograms for stability studies of **18**.
