# Supplementary material for: Identification of Novel Human Breast Carcinoma (MDA-MB-231) Cell Growth Modulators from a Carbohydrate-Based Diversity Oriented Synthesis Library
Source: Molecules. 2016 Oct 20;21(10):1405. doi: 10.3390/molecules21101405 (PMC6273552; doi:10.3390/molecules21101405)
Supplement: Supplementary file 1 [file molecules-21-01405-s001.pdf]

Supplementary Materials: Identification of Novel Human Breast Carcinoma (MDA-MB-231) Cell Growth Modulators from a Carbohydrate-Based Diversity Oriented Synthesis Library

Elena Lenci, Riccardo Innocenti, Alessio Biagioni, Gloria Menchi, Francesca Bianchini and Andrea Trabocchi

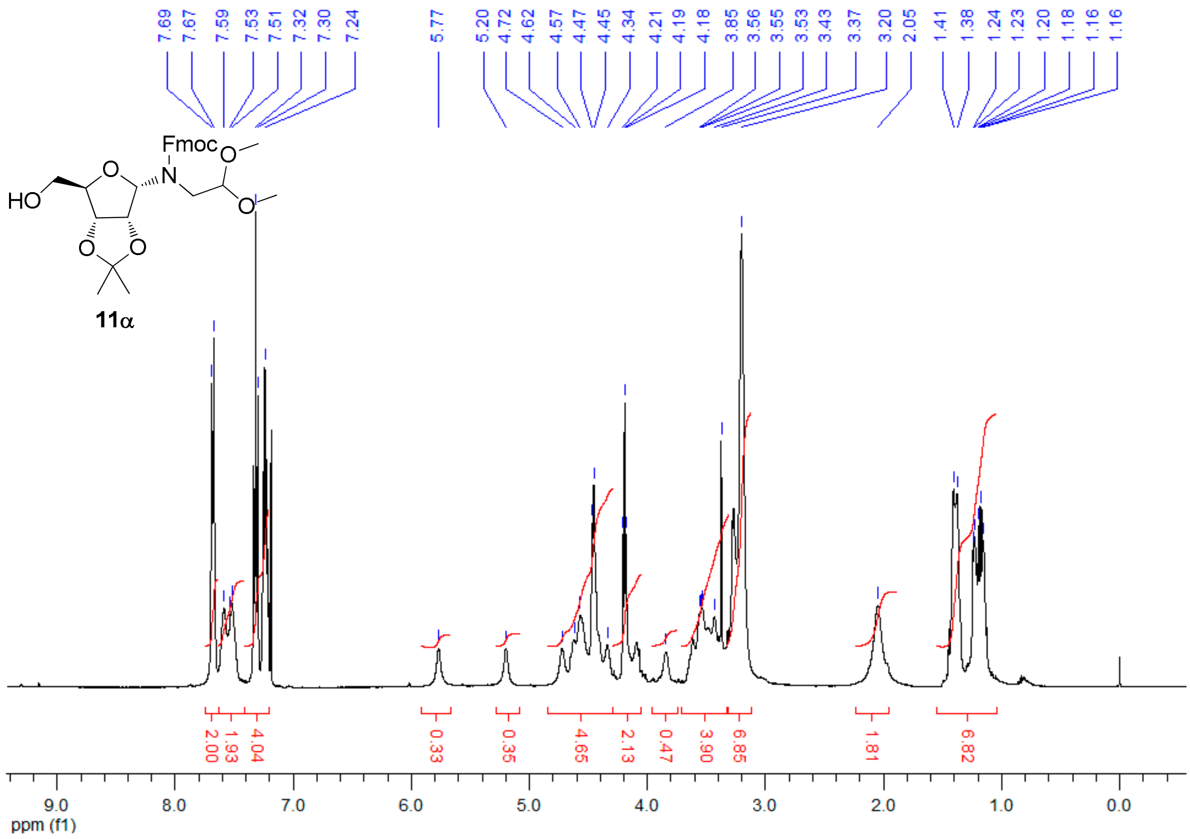

Figure S1. ^{1}H-NMR spectrum of compound **11α** (400 MHz, $CDCl_3$).

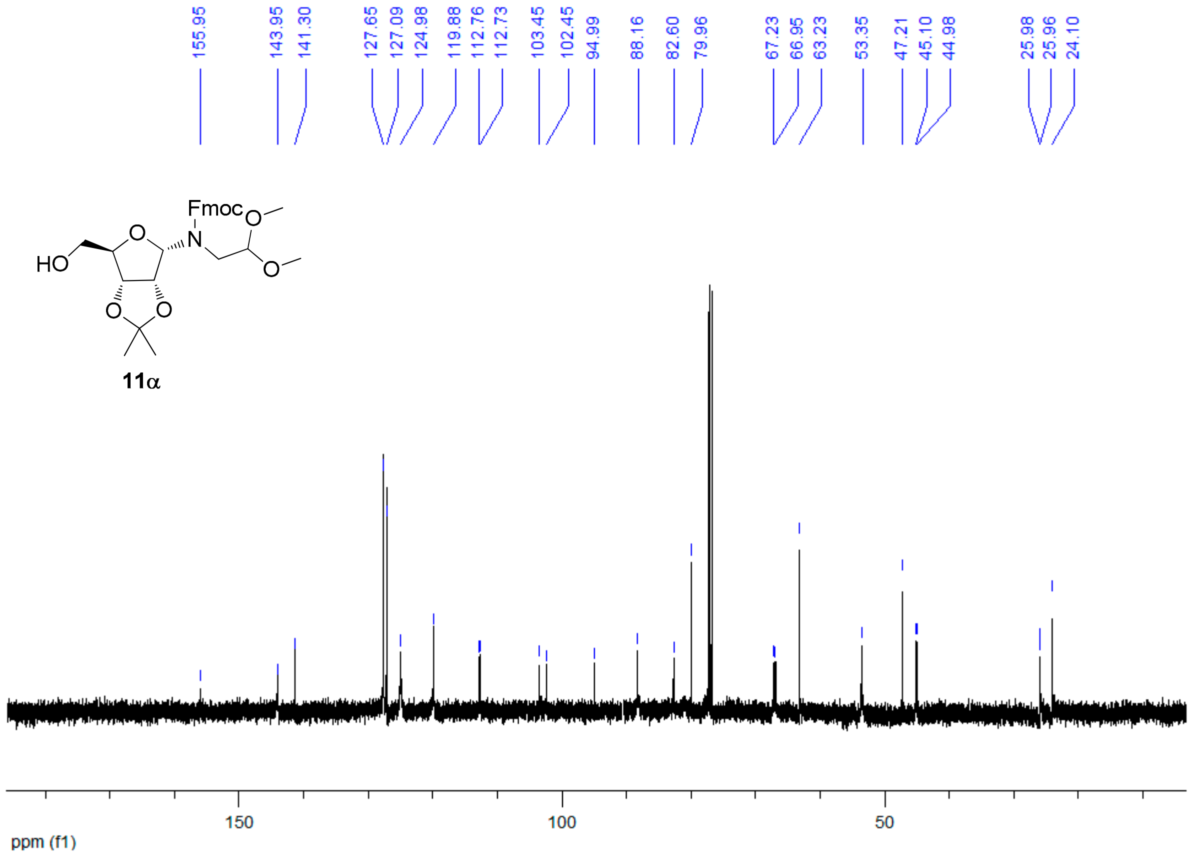

Figure S2. ^{13}C-NMR spectrum of compound **11α** (50 MHz, $CDCl_3$).

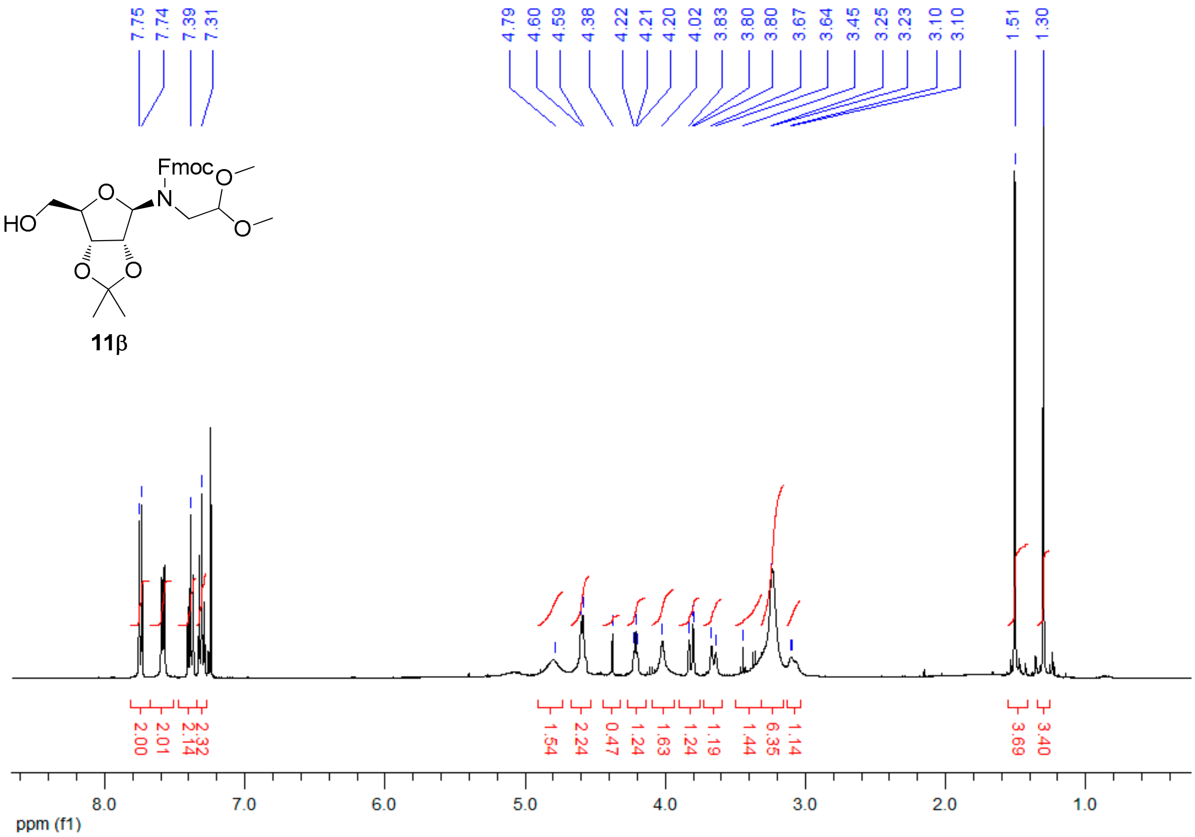

Figure S3. ^{1}H-NMR spectrum of compound **11β** (400 MHz, $CDCl_3$).

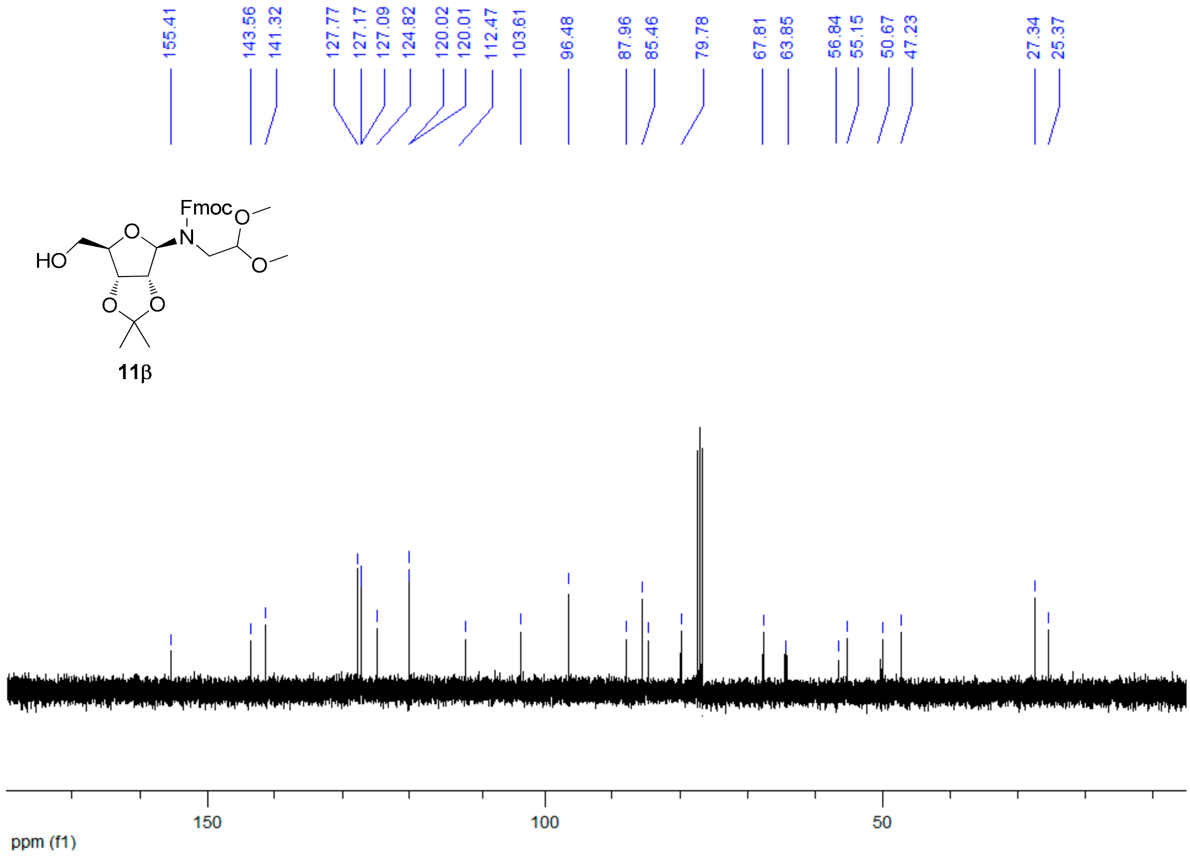

Figure S4. ^{13}C-NMR spectrum of compound **11β** (100 MHz, $CDCl_3$).

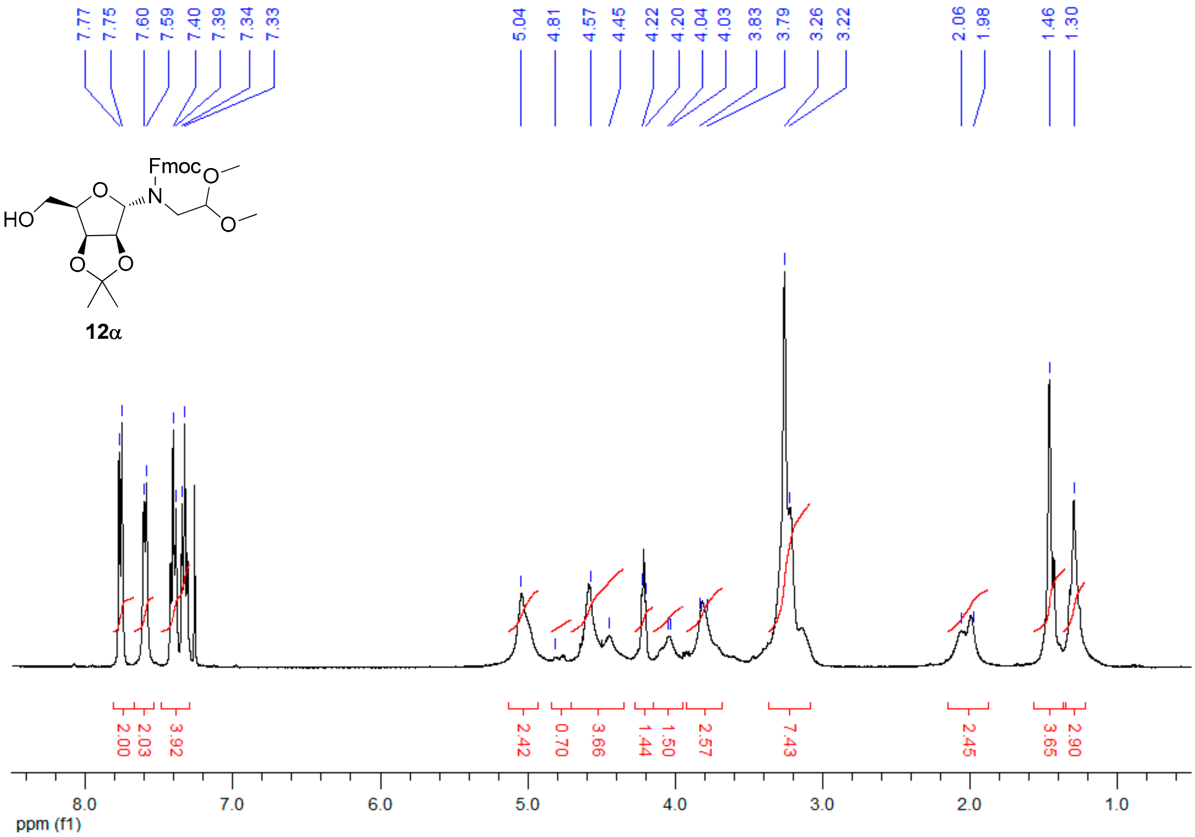

Figure S5. ^{1}H-NMR spectrum of compound **12α** (400 MHz, $CDCl_3$).

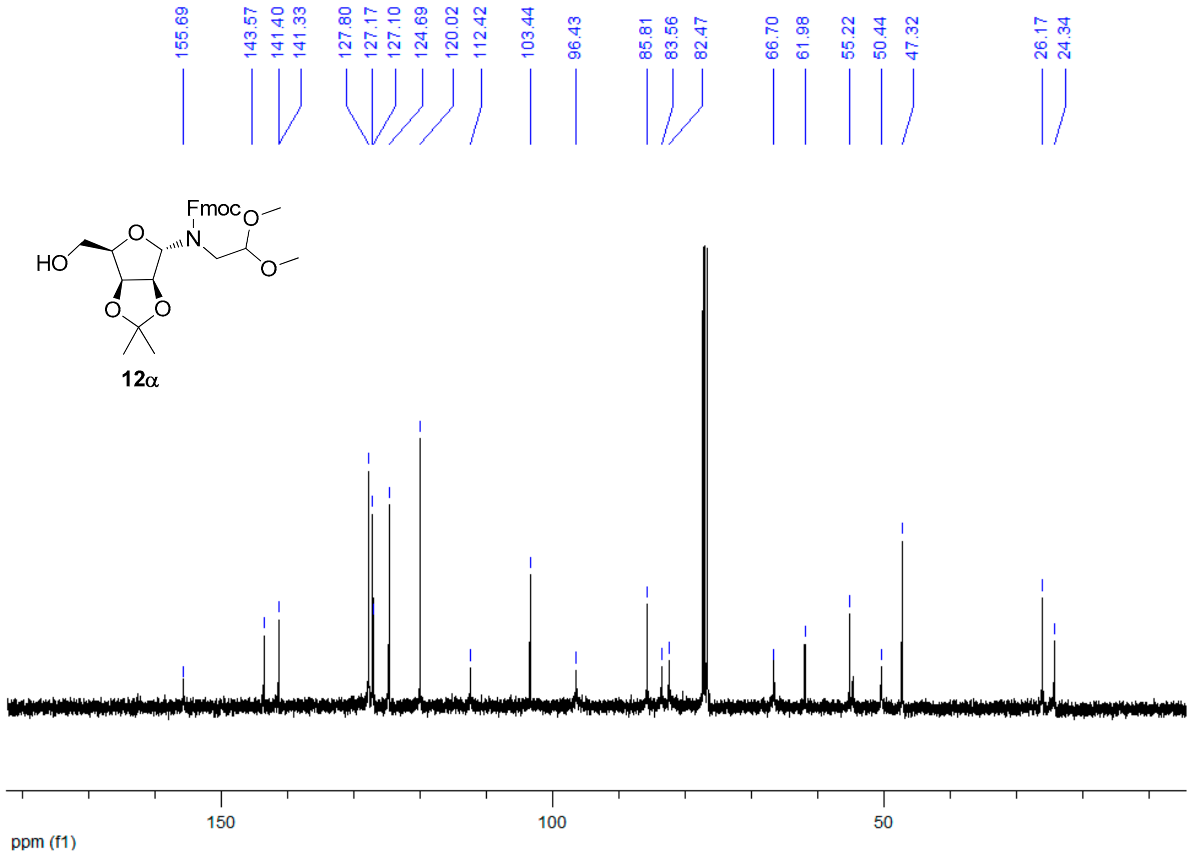

Figure S6. ^{13}C-NMR spectrum of compound **12α** (50 MHz, $CDCl_3$).

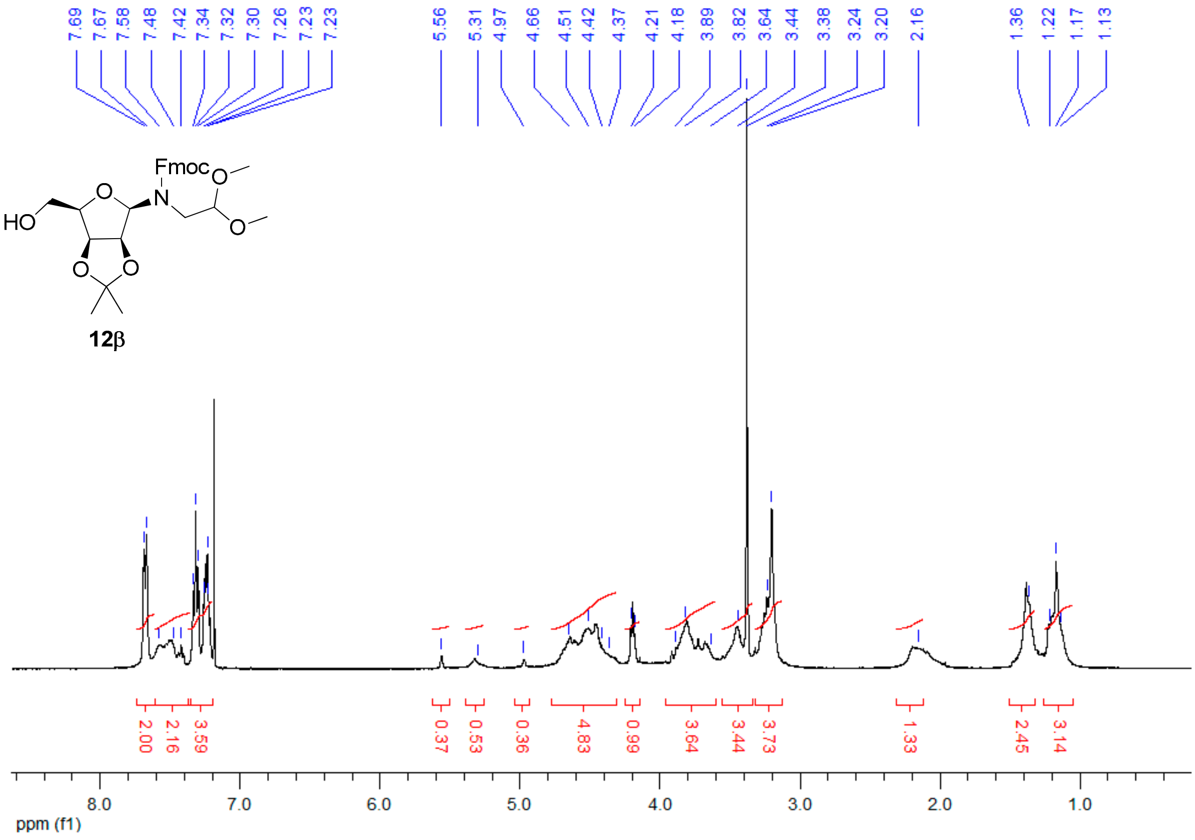

Figure S7. ^{1}H-NMR spectrum of compound **12β** (400 MHz, $CDCl_3$).

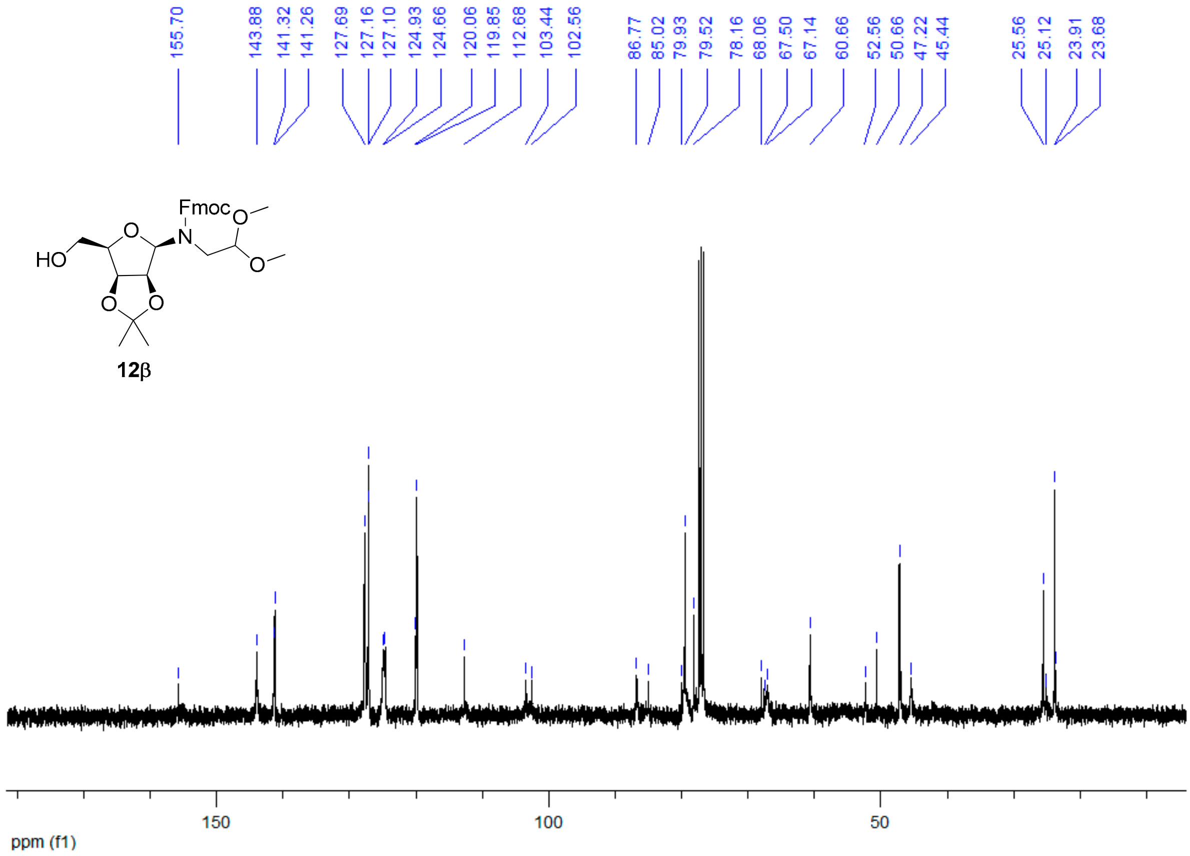

Figure S8. ^{13}C-NMR spectrum of compound **12β** (100 MHz, $CDCl_3$).

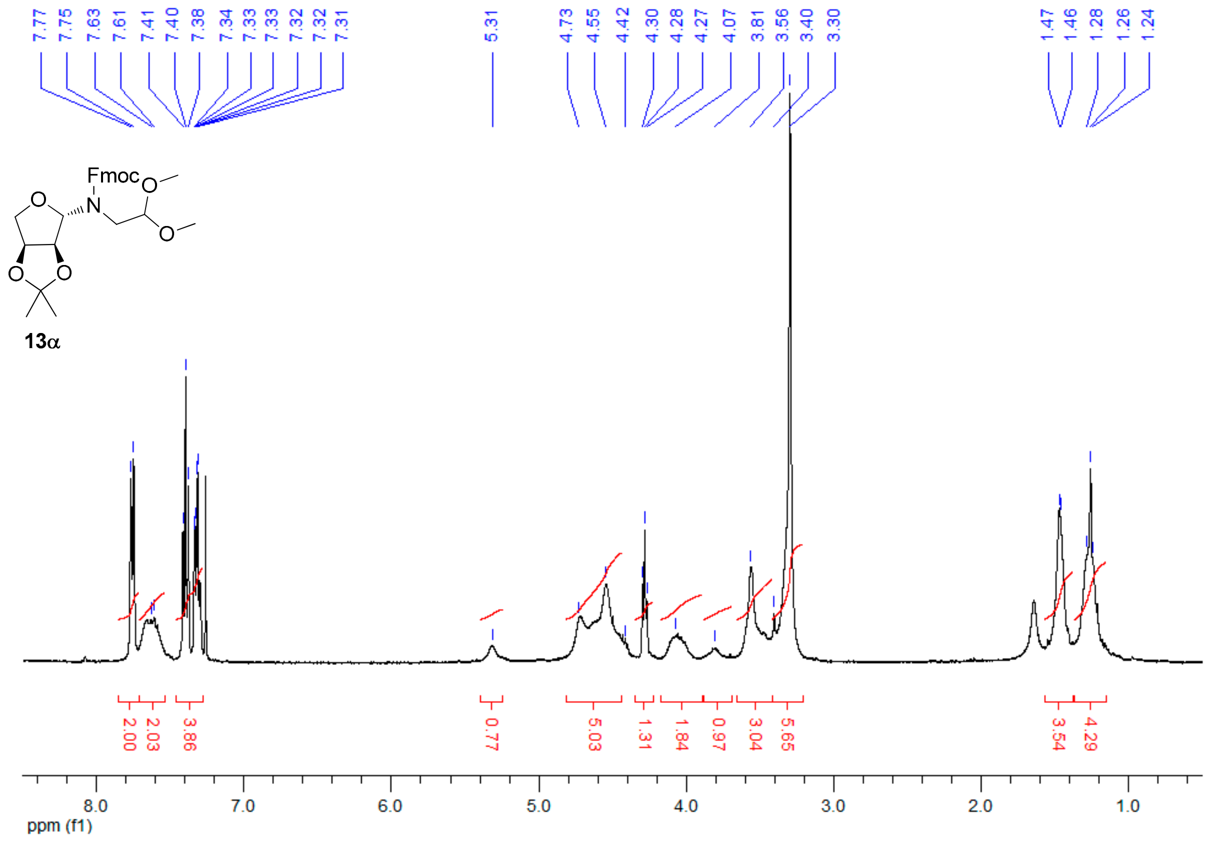

Figure S9. ^{1}H-NMR spectrum of compound **13α** (400 MHz, $CDCl_3$).

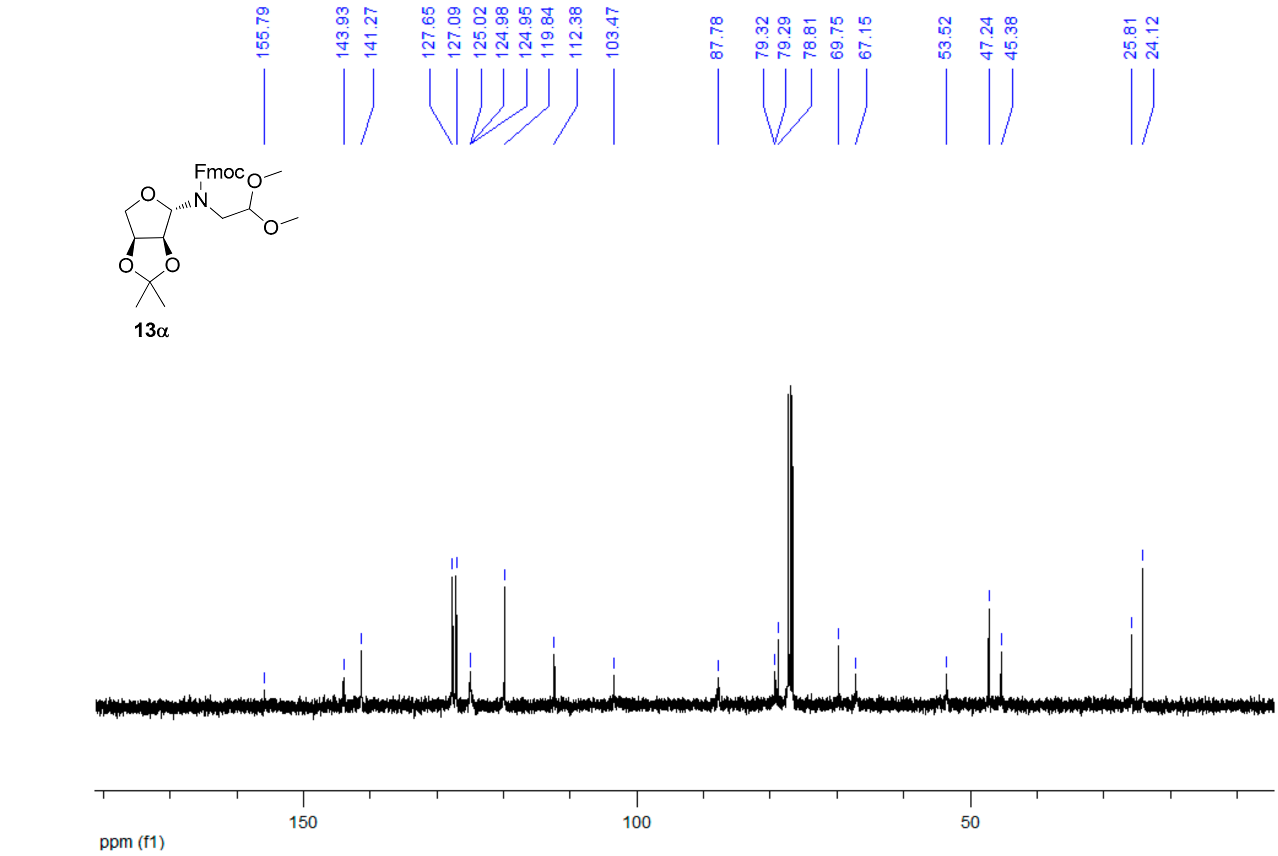

Figure S10. ^{13}C-NMR spectrum of compound **13*α*** (100 MHz, $CDCl_3$).

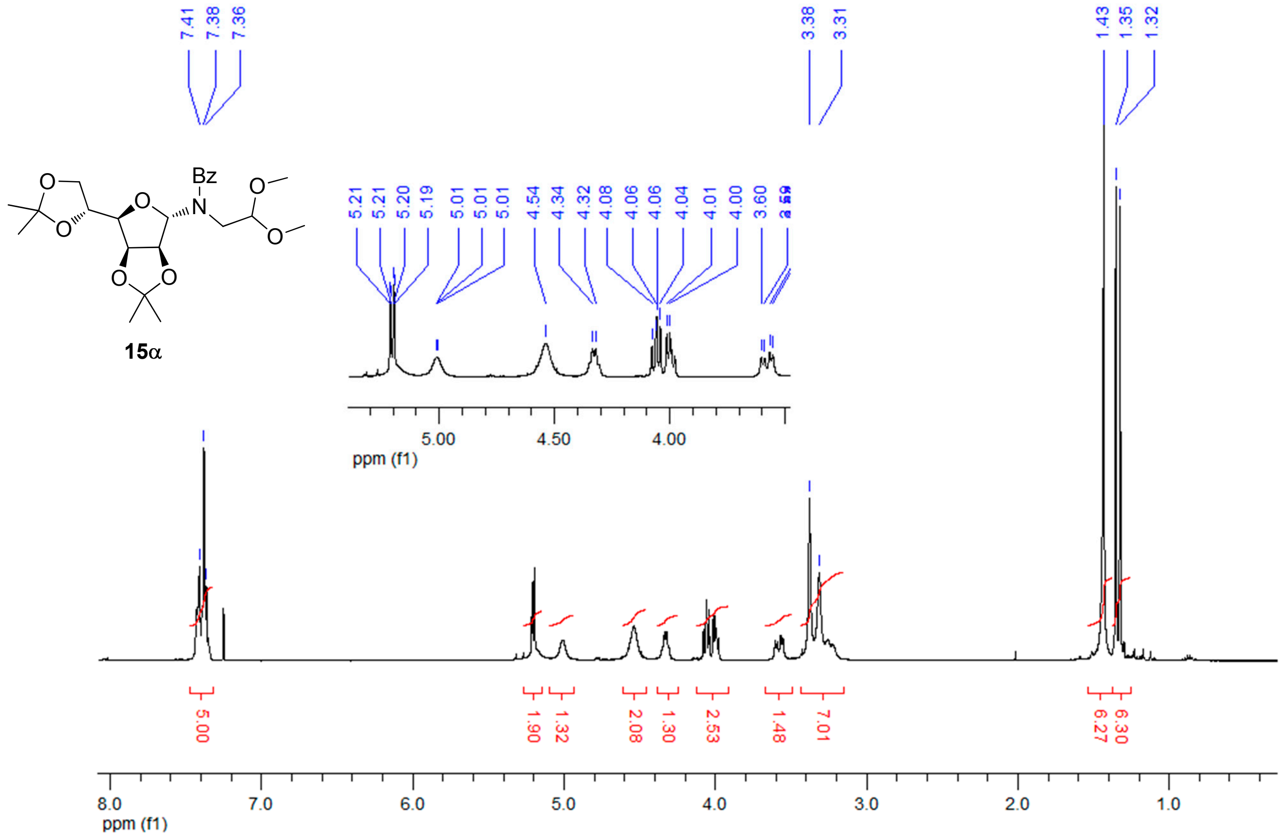

Figure S11. ^{1}H-NMR spectrum of compound **15*α*** (400 MHz, $CDCl_3$).

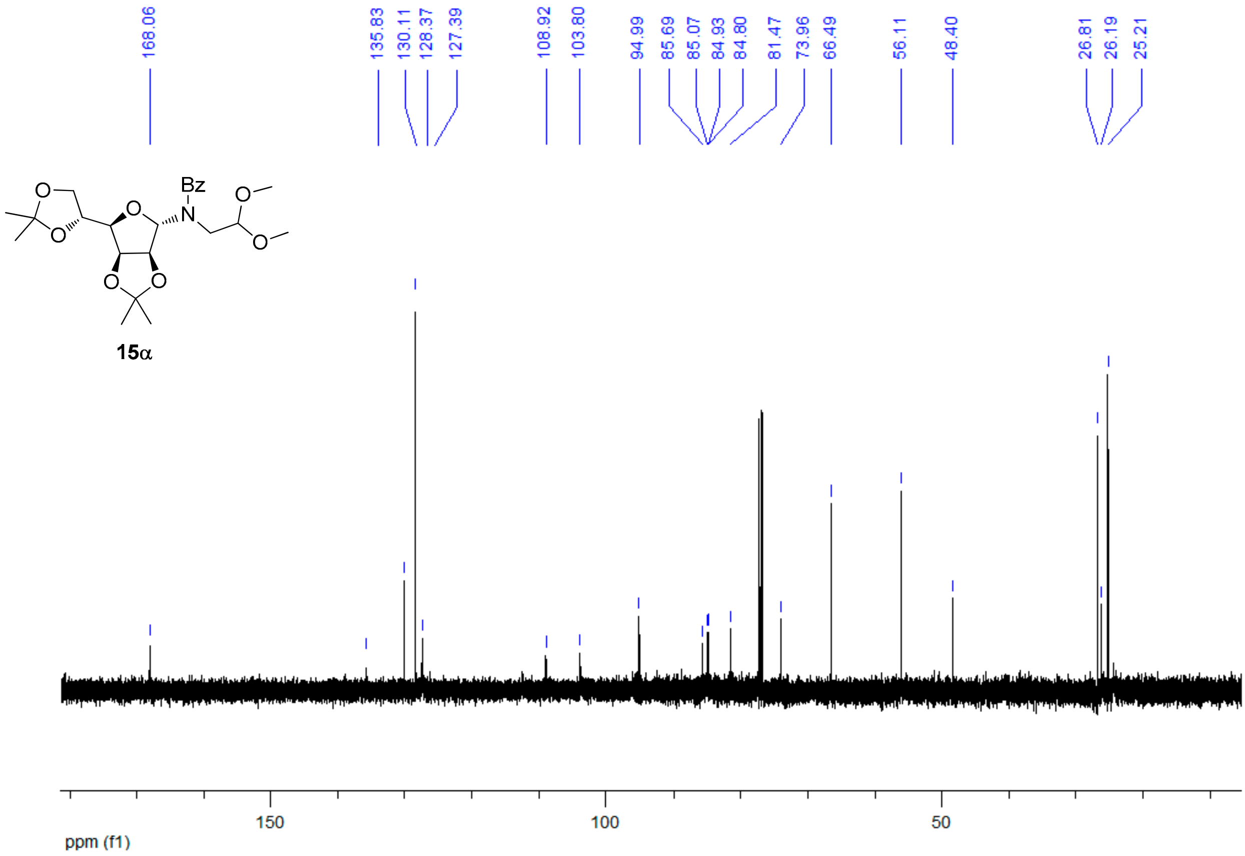

Figure S12. ^{13}C-NMR spectrum of compound **15α** (100 MHz, $CDCl_3$).

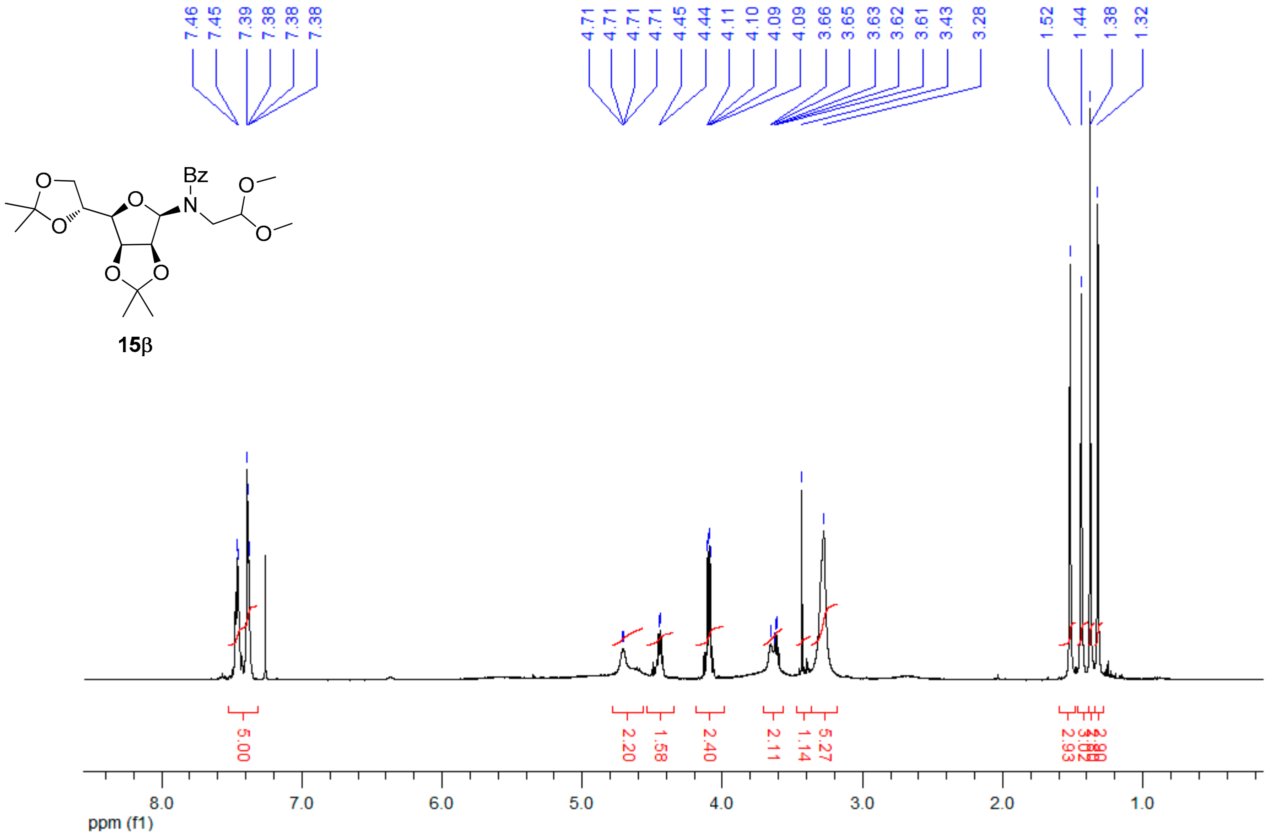

Figure S13. ^{1}H-NMR spectrum of compound **15β** (400 MHz, $CDCl_3$).

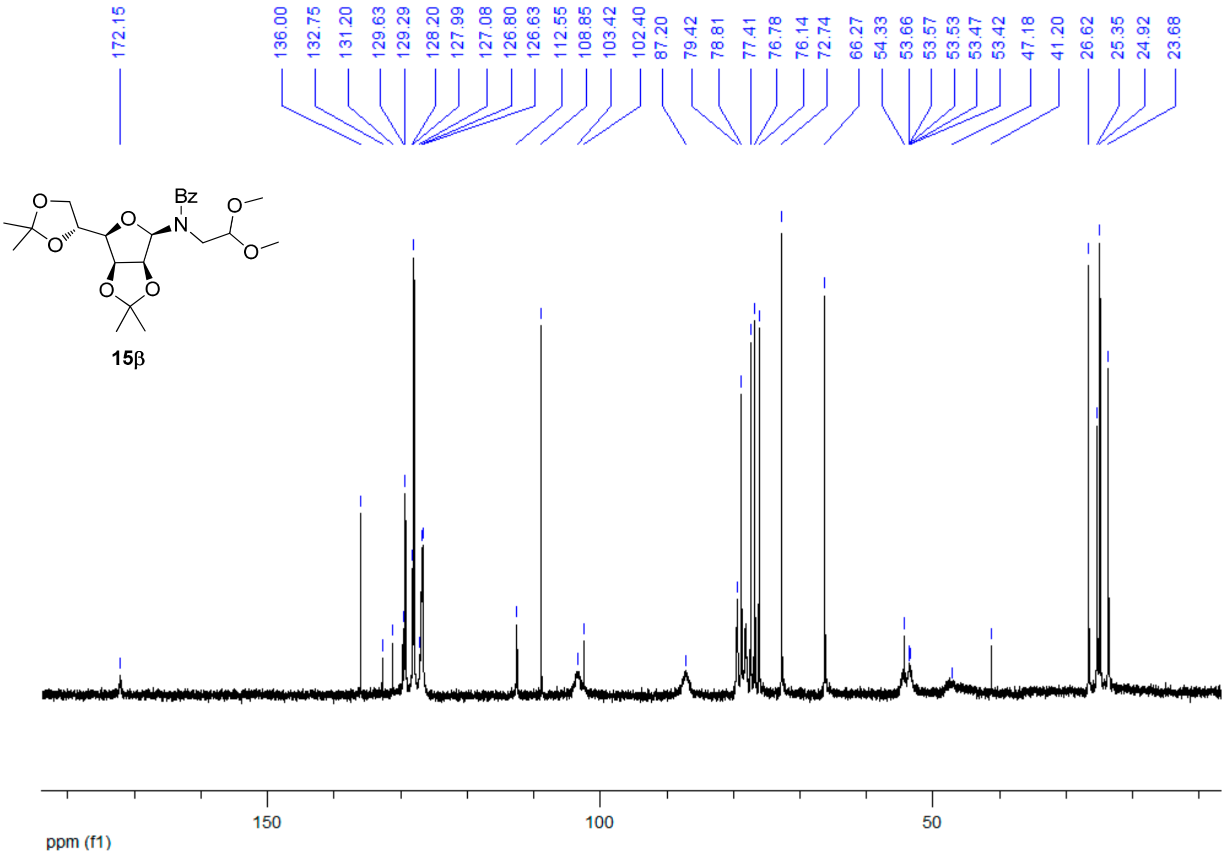

Figure S14. ^{13}C-NMR spectrum of compound **15β** (100 MHz, CDCl$_3$).

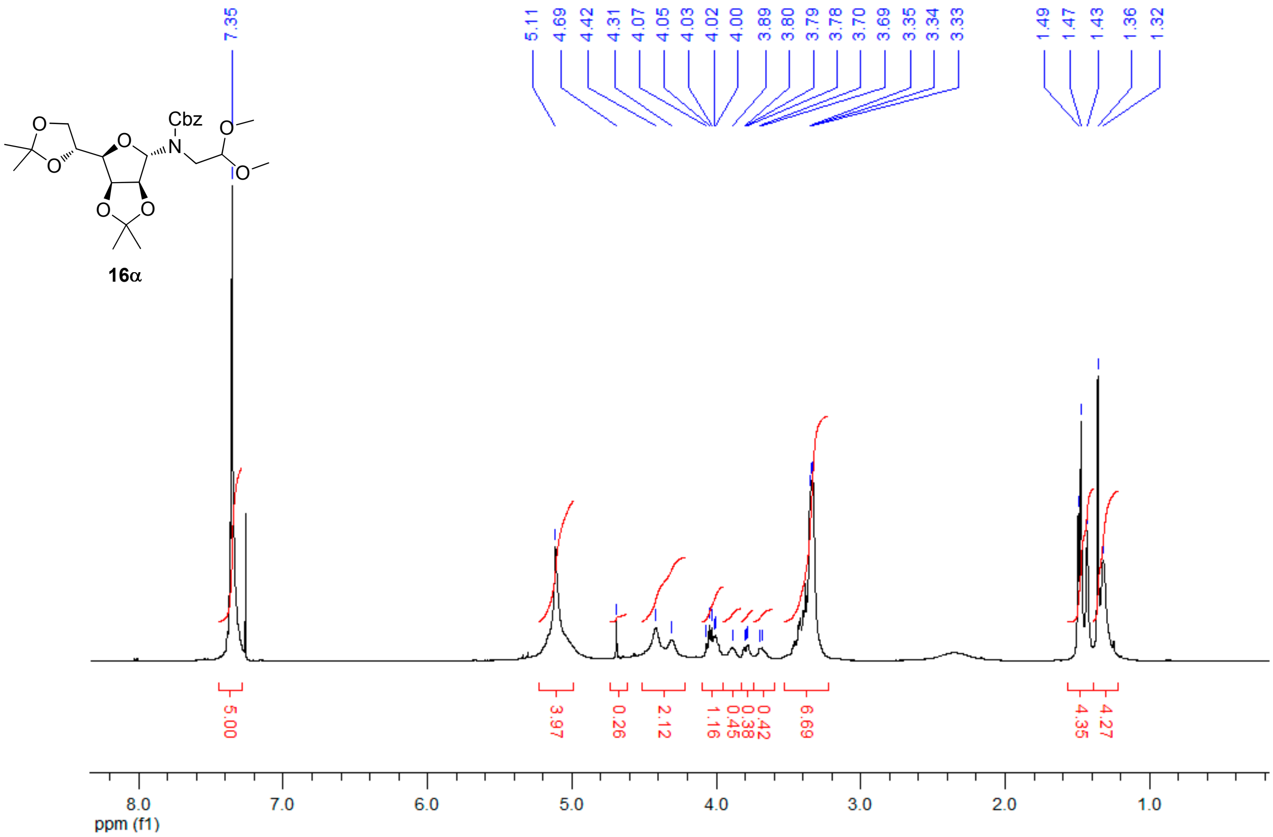

Figure S15. ^{1}H-NMR spectrum of compound **16α** (400 MHz, CDCl$_3$).

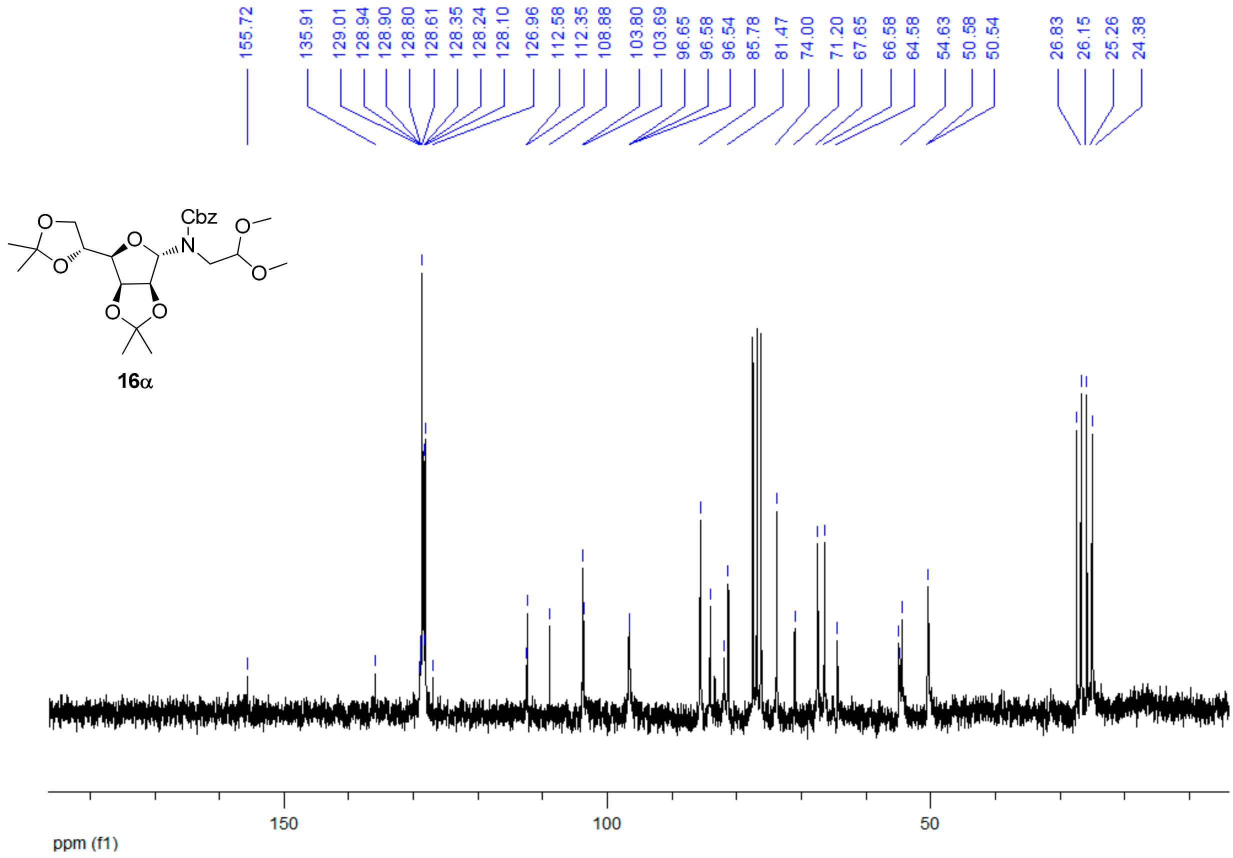

Figure S16. ^{13}C-NMR spectrum of compound **16α** (50 MHz, $CDCl_3$).

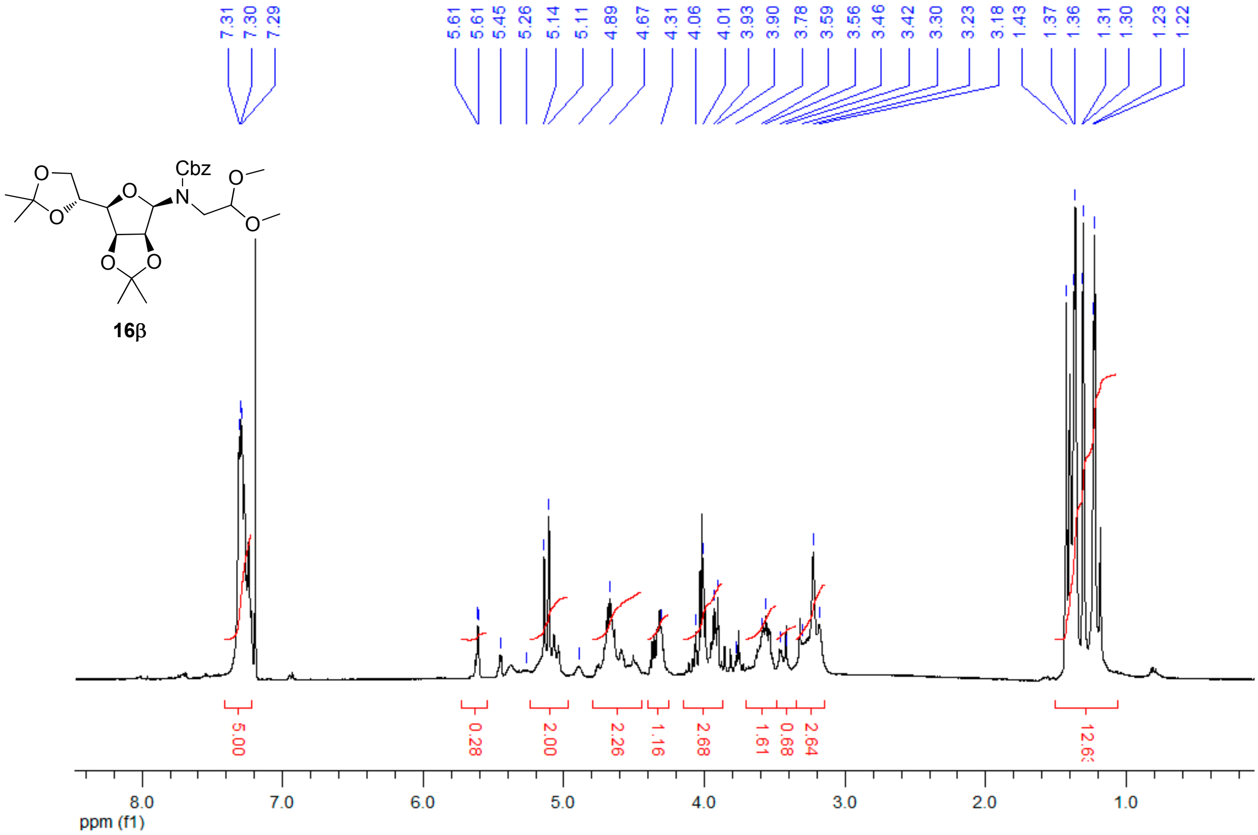

Figure S17. ^{1}H-NMR spectrum of compound **16β** (400 MHz, $CDCl_3$).

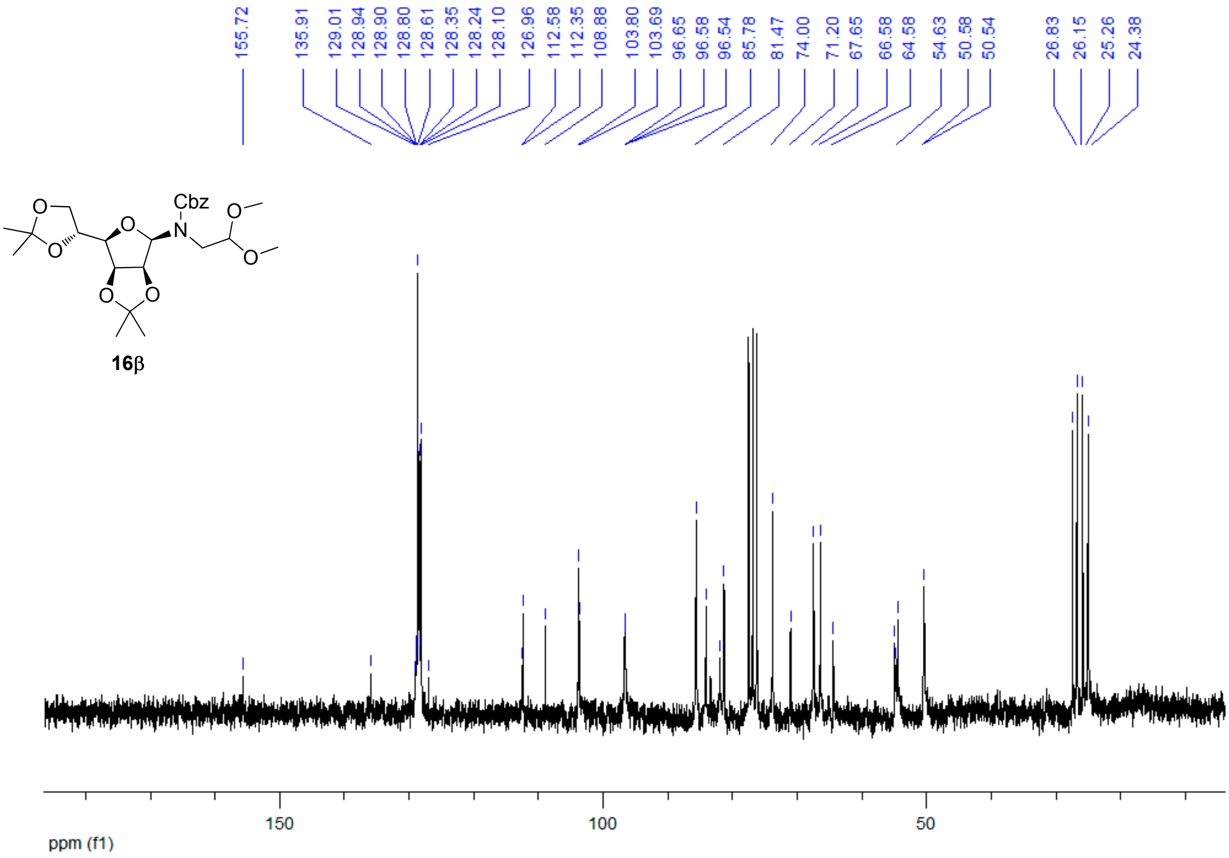

Figure S18. ^{13}C-NMR spectrum of compound **16β** (50 MHz, $CDCl_3$).

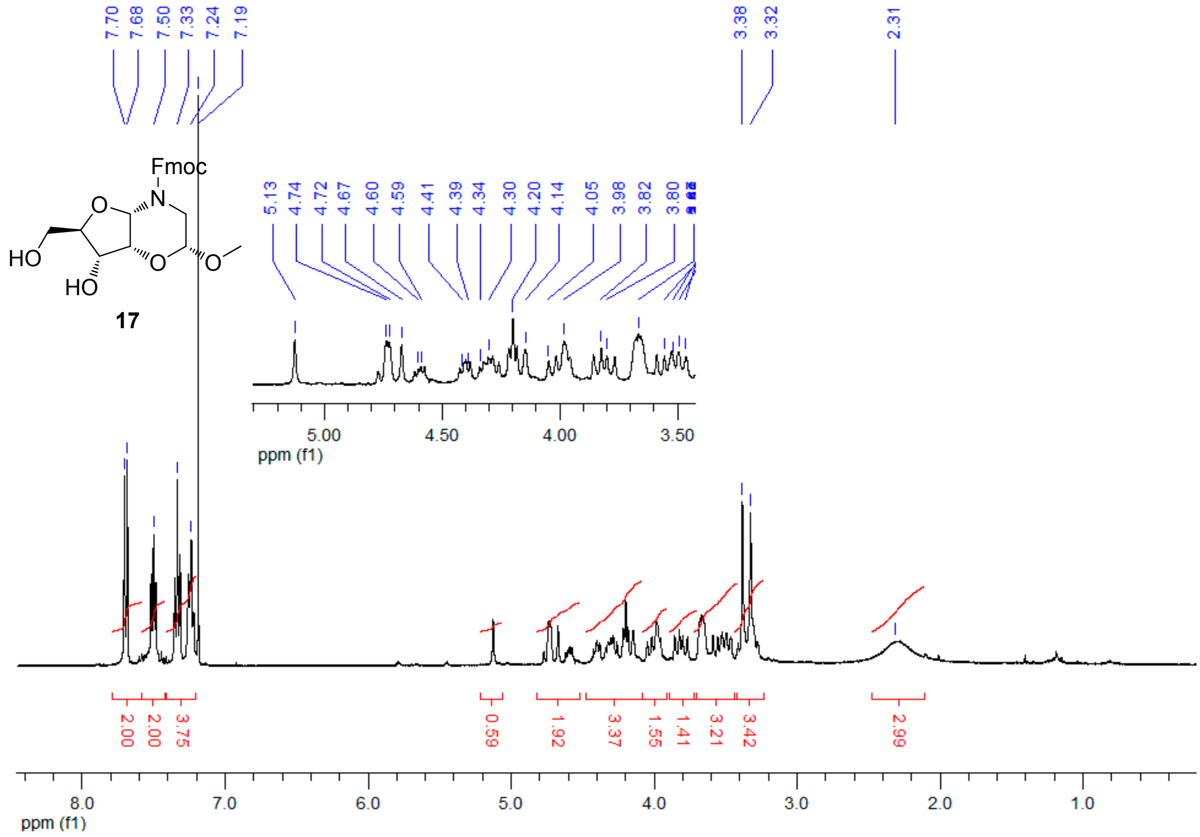

Figure S19. ^{1}H-NMR spectrum of compound **17** (400 MHz, $CDCl_3$).

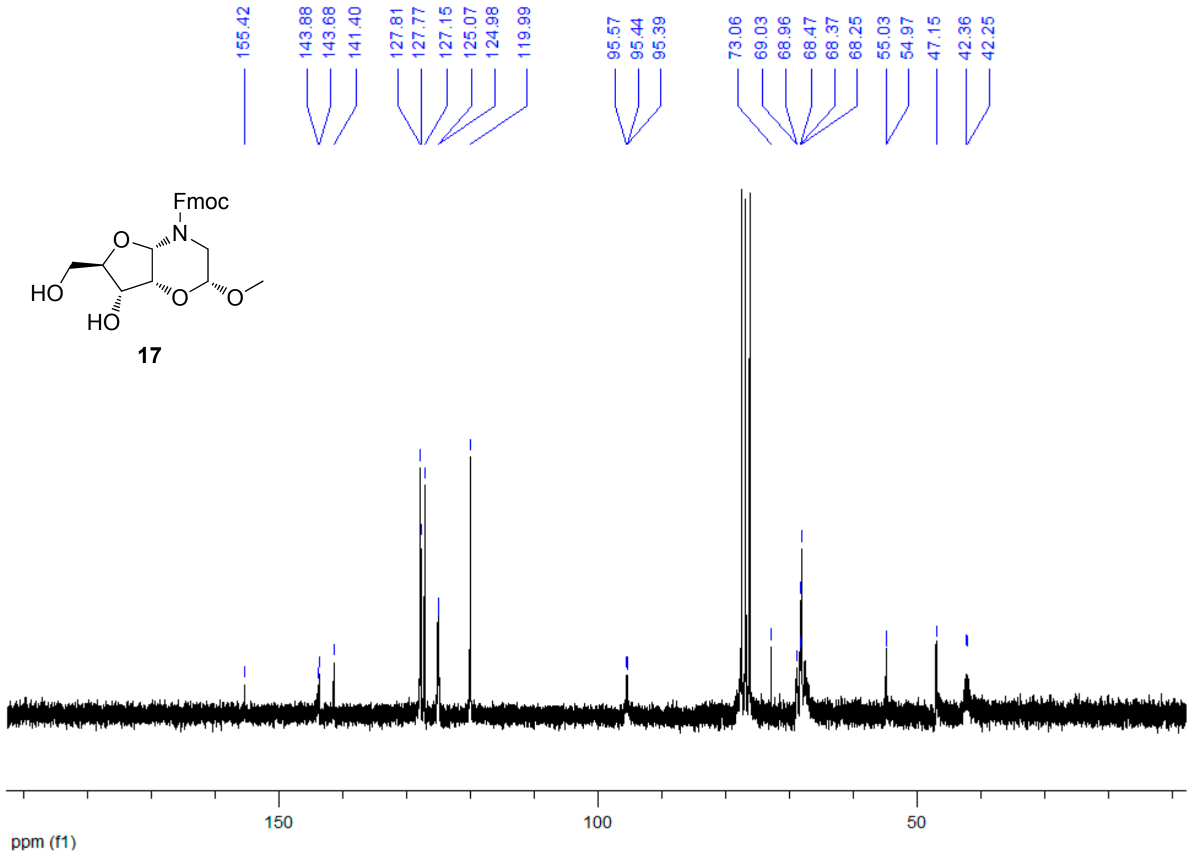

Figure S20. ^{13}C-NMR spectrum of compound **17** (100 MHz, $CDCl_3$).

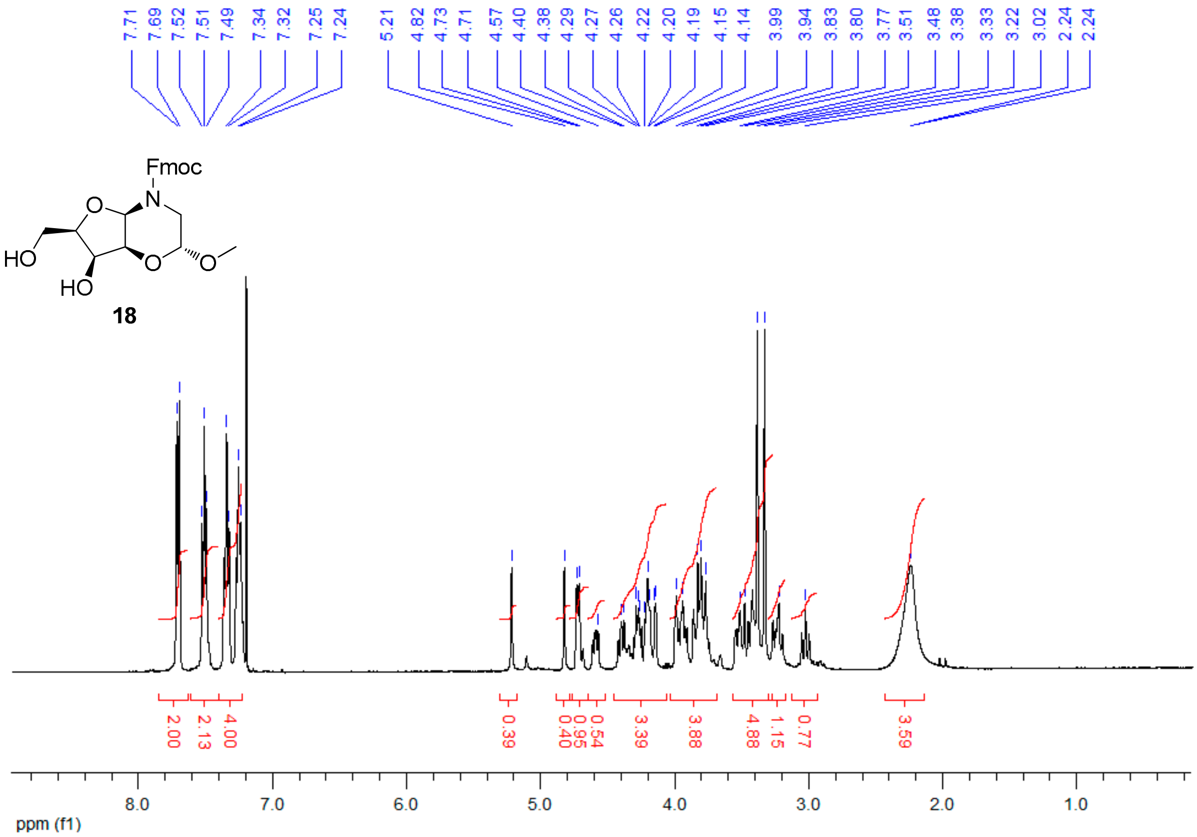

Figure S21. ^{1}H-NMR spectrum of compound **18** (400 MHz, $CDCl_3$).

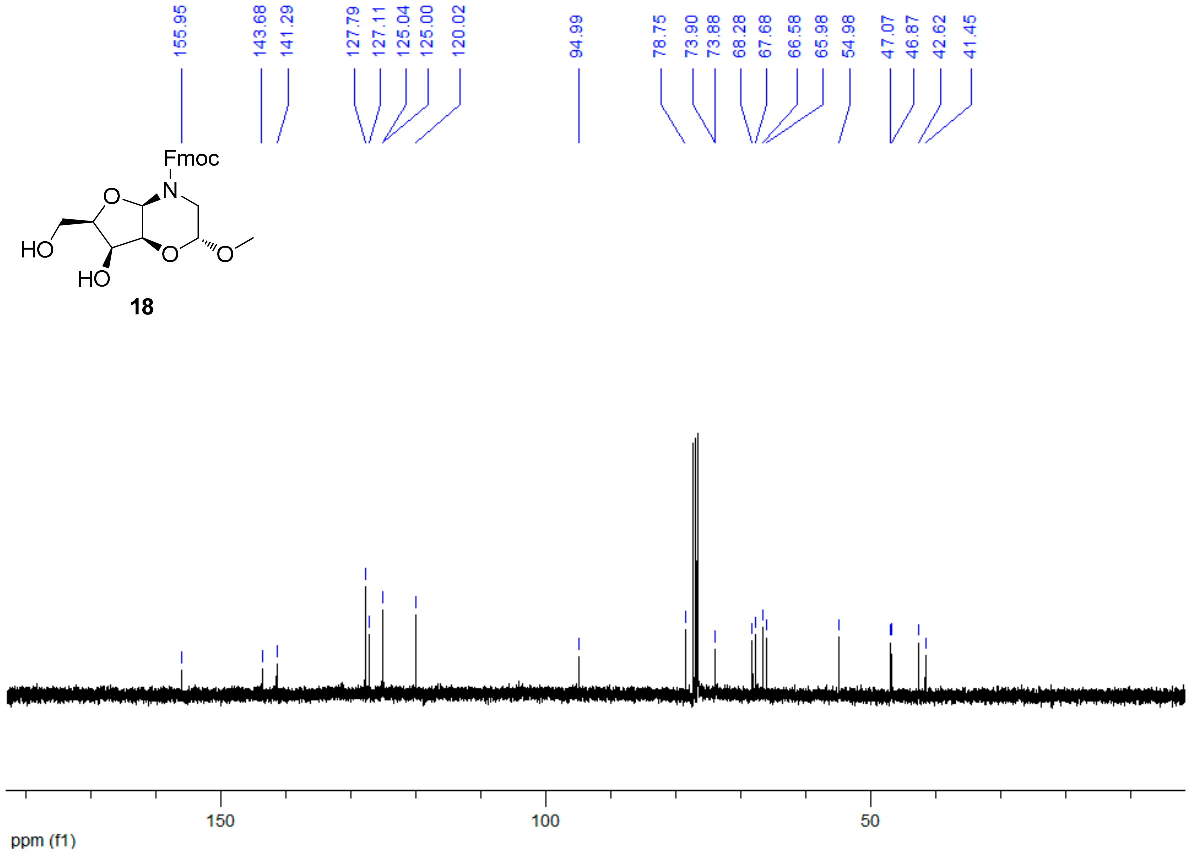

Figure S22. ^{13}C-NMR spectrum of compound **18** (100 MHz, $CDCl_3$).

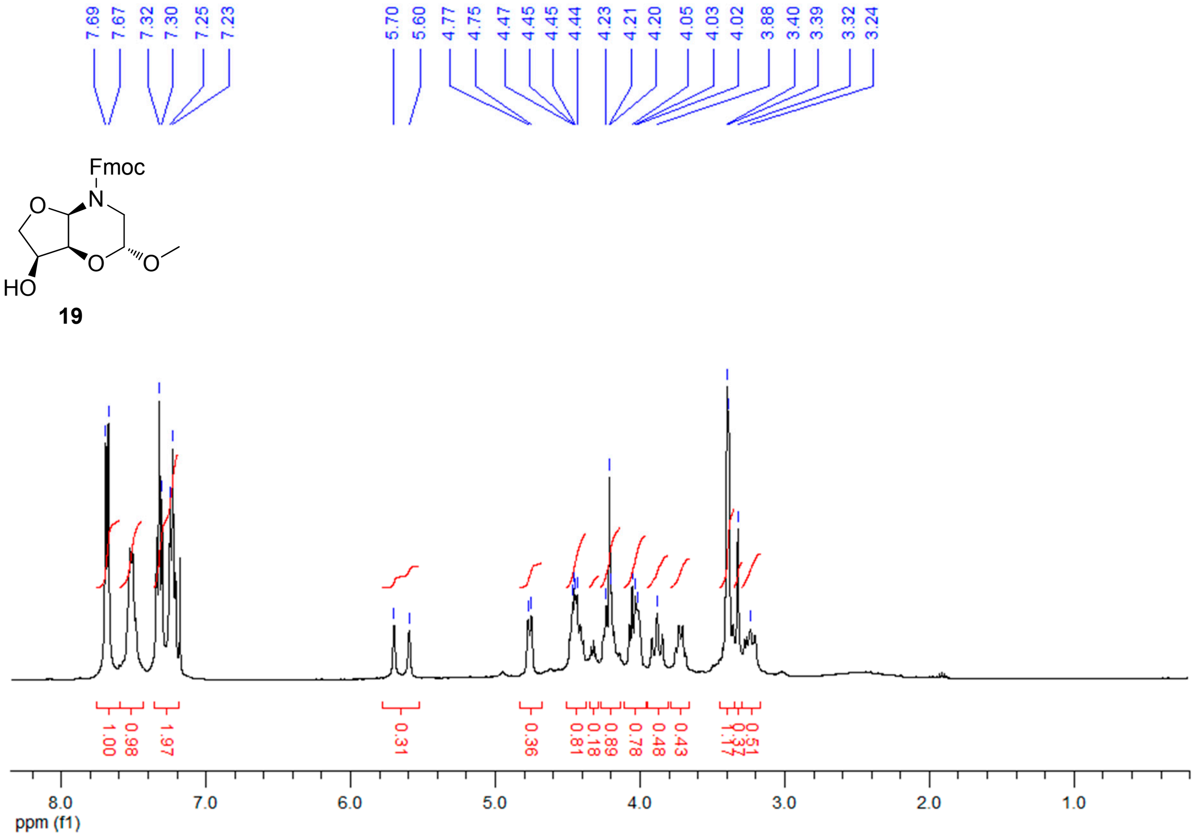

Figure S23. ^{1}H-NMR spectrum of compound **19** (400 MHz, $CDCl_3$).

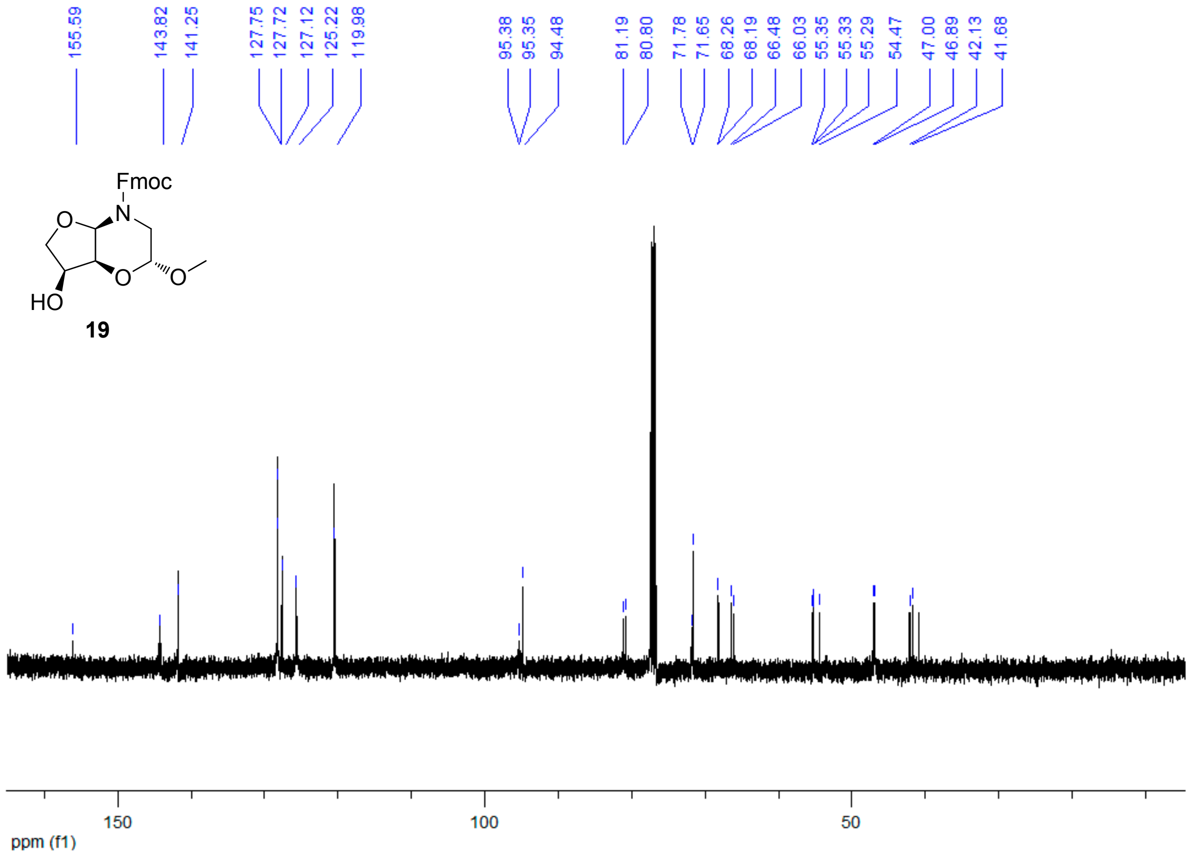

Figure S24. ^{13}C-NMR spectrum of compound **19** (100 MHz, $CDCl_3$).

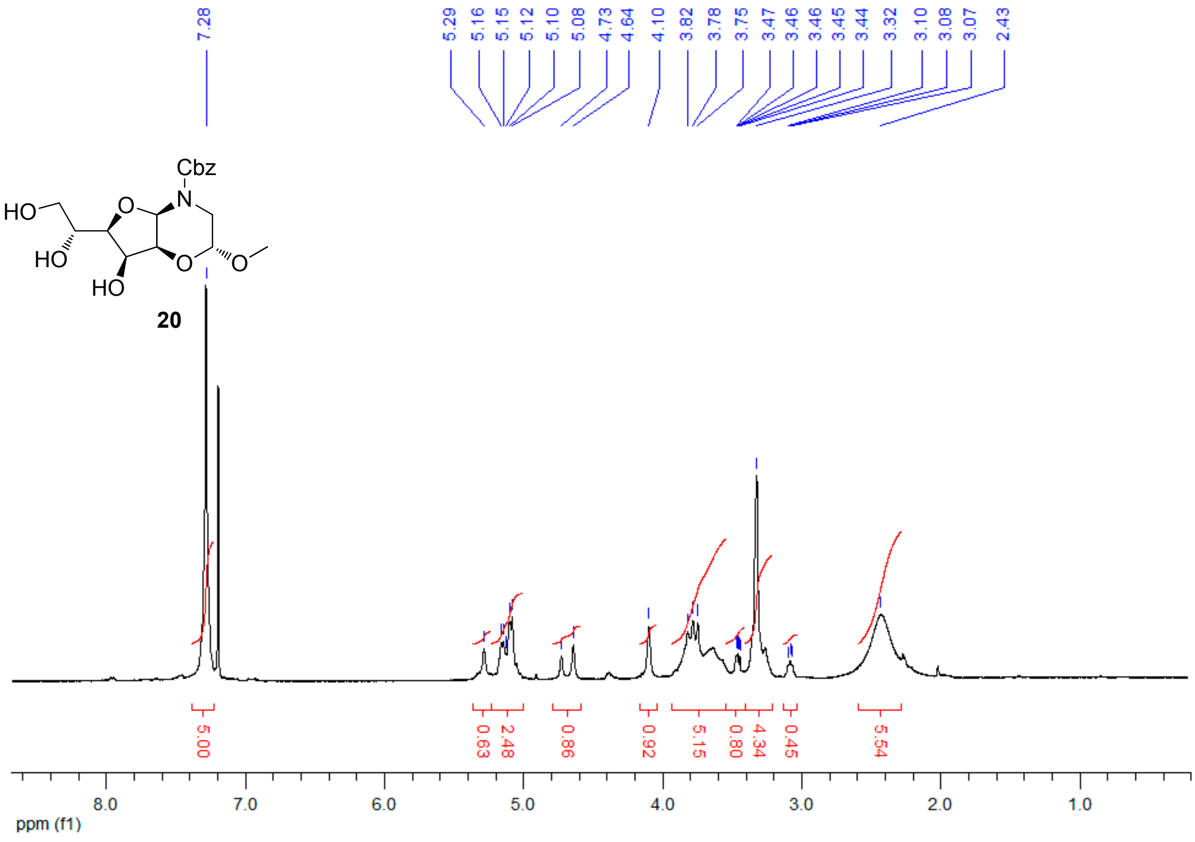

Figure S25. ^{1}H-NMR spectrum of compound **20** (400 MHz, $CDCl_3$).

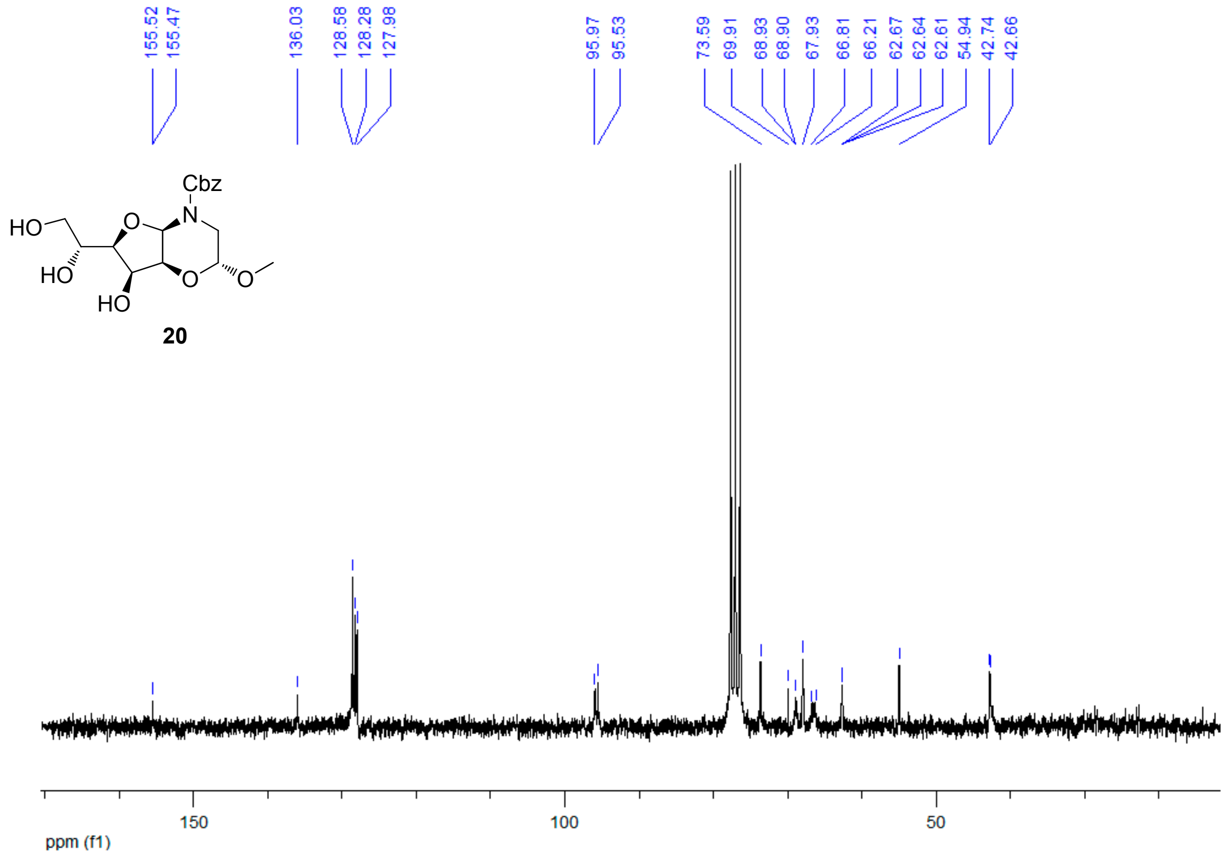

Figure S26. ^{13}C-NMR spectrum of compound **20** (50 MHz, $CDCl_3$).

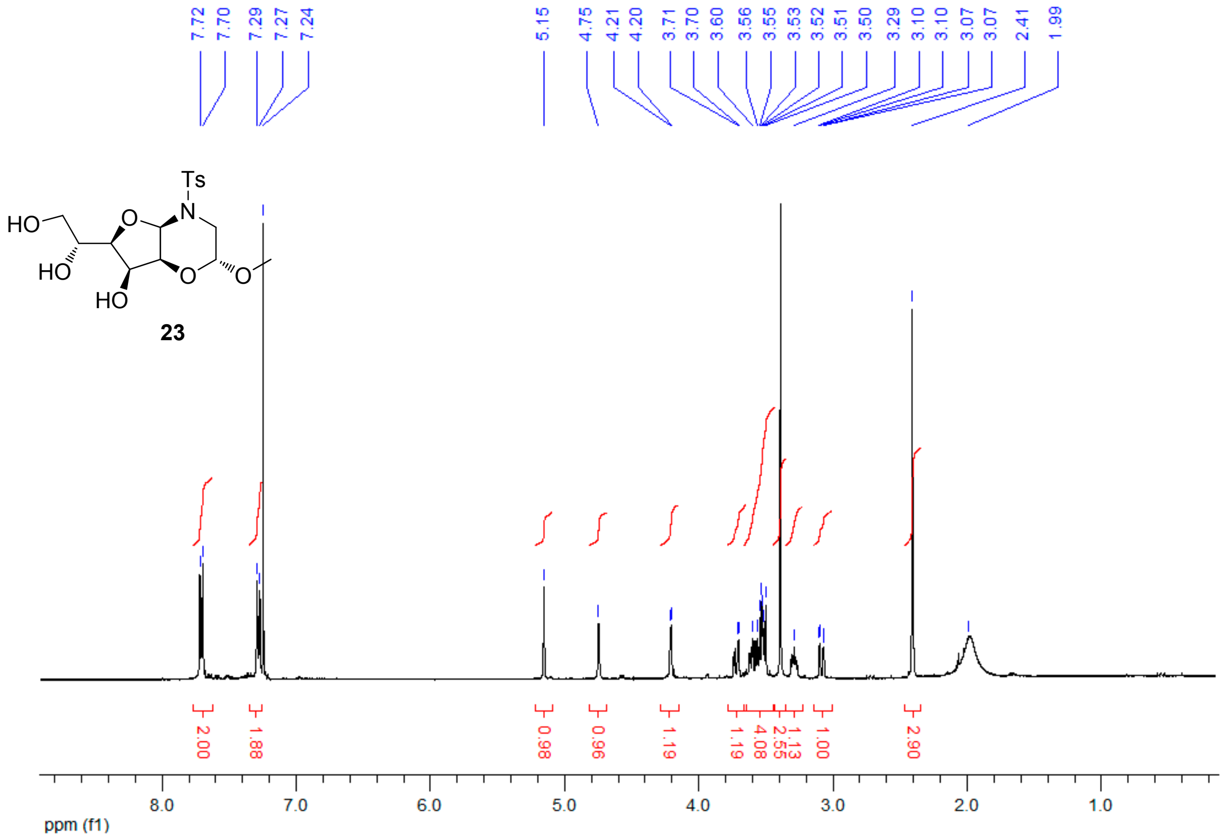

Figure S27. ^{1}H-NMR spectrum of compound **23** (400 MHz, $CDCl_3$).

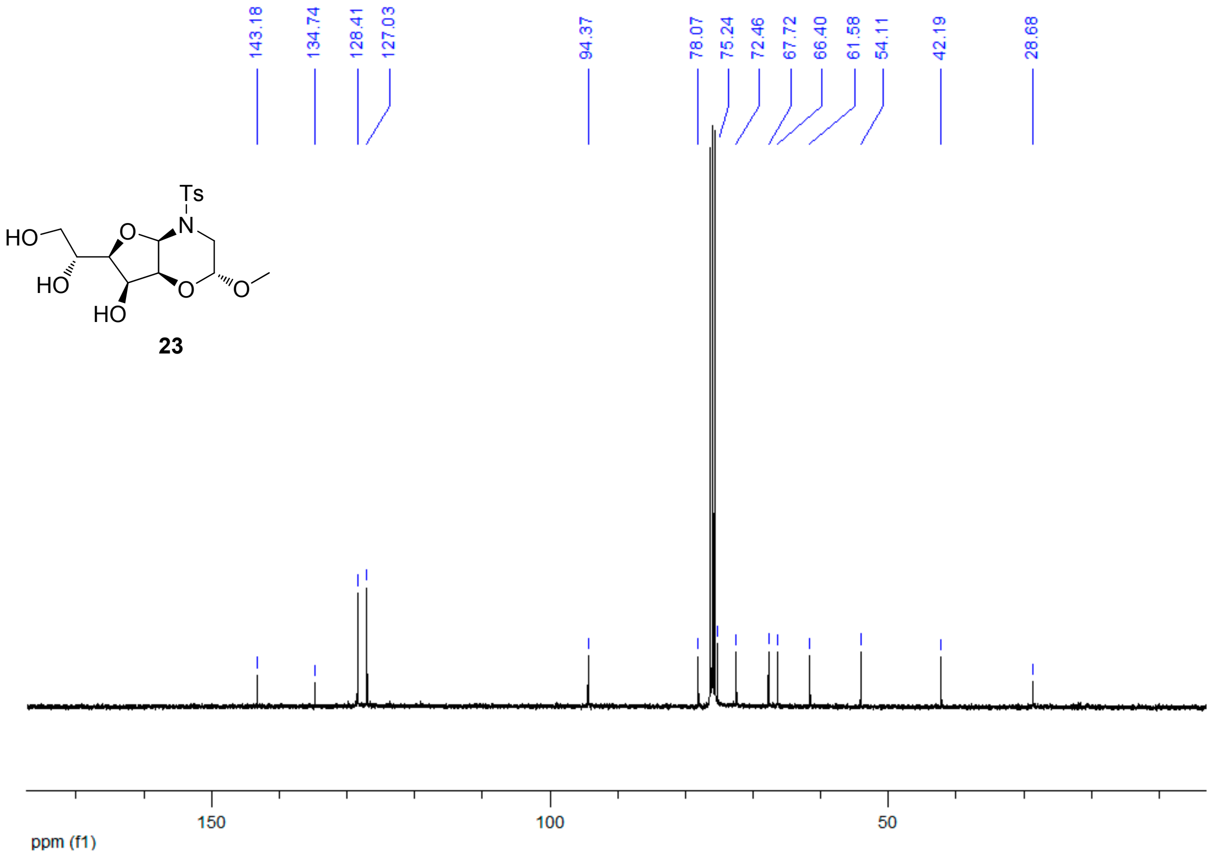

Figure S28. ^{13}C-NMR spectrum of compound **23** (100 MHz, $CDCl_3$).

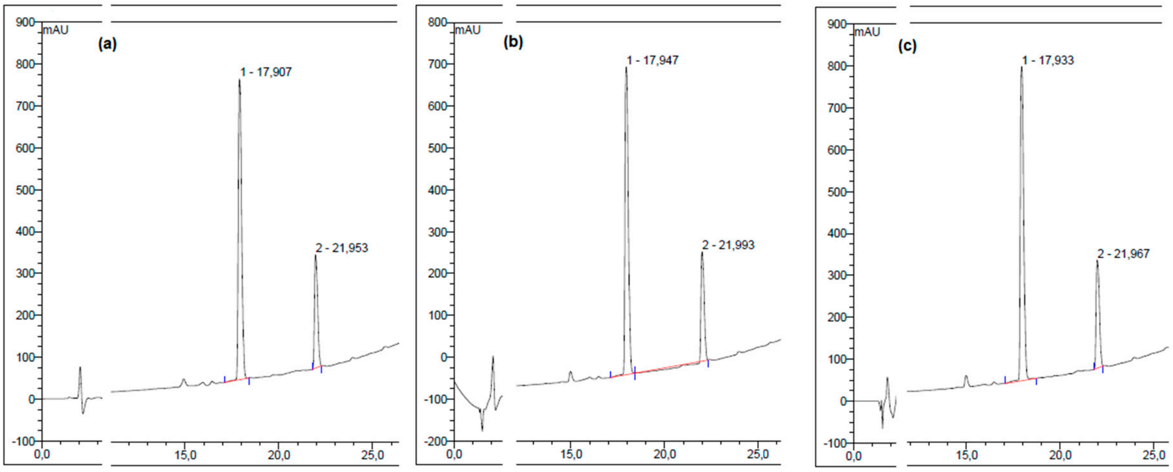

Figure S29. HPLC runs of compound **18** (0.5 mg/mL, final conc.) after 1h incubation in (a) CH_3CN; (b) 1:1 mixture of CH_3CN and HCl 1M; (c) 1:1 mixture of MeOH and HCl 1M. A 0.1 mg/mL quantity (final conc.) of benzophenone (rt = 22 min) was used as an internal standard.

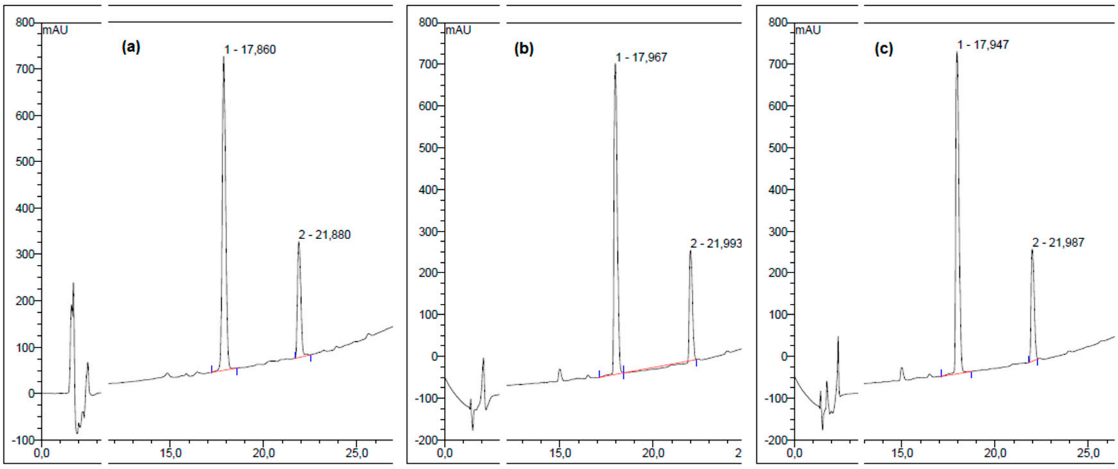

Figure S30. HPLC runs of compound **18** (0.5 mg/mL, final conc.) after 24h incubation in (a) CH_3CN; (b) 1:1 mixture of CH_3CN and HCl 1M; (c) 1:1 mixture of MeOH and HCl 1M. A 0.1 mg/mL quantity (final conc.) of benzophenone (rt = 22 min) was used as an internal standard.
